# Seasonality in Spatial Turnover of Bacterioplankton Along an Ecological Gradient in the East China Sea: Biogeographic Patterns, Processes and Drivers

**DOI:** 10.3390/microorganisms8101484

**Published:** 2020-09-27

**Authors:** Hanjing Hu, Jiaying He, Huizhen Yan, Dandi Hou, Demin Zhang, Lian Liu, Kai Wang

**Affiliations:** 1 State Key Laboratory for Managing Biotic and Chemical Threats to the Quality and Safety of Agro-Products, Ningbo University, Ningbo 315211, China; hhj2141@163.com (H.H.); zhangdemin@nbu.edu.cn (D.Z.); 2 School of Marine Sciences, Ningbo University, Ningbo 315211, China; joyhejiaying@outlook.com (J.H.); hzyan1995@163.com (H.Y.); houdandi@nbu.edu.cn (D.H.); 3 Collaborative Innovation Center for Zhejiang Marine High-Efficiency and Healthy Aquaculture, Ningbo 315211, China; 4 Marine Environmental Monitoring Center of Ningbo, SOA, Ningbo 315012, China

**Keywords:** bacterioplankton, spatial turnover, seasonality, community assembly, biogeographic pattern

## Abstract

Seasonal succession in bacterioplankton is a common process in marine waters. However, seasonality in their spatial turnover is largely unknown. Here, we investigated spatial turnover of surface bacterioplankton along a nearshore-to-offshore gradient in the East China Sea across four seasons. Although seasonality overwhelmed spatial variability of bacterioplankton composition, we found significant spatial turnover of bacterioplankton along the gradient as well as overall seasonal consistency in biogeographic patterns (including distance–decay relationship and covariation of community composition with distance to shore) with subtle changes. Bacterioplankton assembly was consistently dominated by deterministic mechanisms across seasons, with changes in specific processes. We found overall seasonal consistency in abiotic factors (mainly salinity and nitrogen and phosphorus nutrients) shaping bacterioplankton composition, while phytoplankton showed a similar influence as abiotic factors only in spring. Although key taxa responsible for bacterioplankton spatial turnover showed certain season-specificity, seasonal switching between closely related taxa occurred within most dominant families. Moreover, many close relatives showed different responding patterns to the environmental gradients in different seasons, suggesting their differences in both seasonally climatic and spatially environmental preferences. Our results provide insights into seasonal consistency and variability in spatial turnover of bacterioplankton in terms of biogeographic patterns, ecological processes, and external and internal drivers.

## 1. Introduction

Marine bacterioplankton are important contributors to biogeochemical cycles, and understanding their spatiotemporal variability and underlying mechanisms is fundamental in unveiling how they are functioning across space and time [1]. A large number of studies, based on the long-term observations in certain stations such as San Pedro Ocean Time-series station (SPOT), Bermuda Atlantic Time-series study (BATS), and Western English Channel (L4), have demonstrated the ubiquity of seasonality and/or annual recurrent patterns in diversity and composition of bacterioplankton communities across global marine ecosystems [2,3,4,5,6,7], with higher dynamics in the surface waters compared with the deeper waters [8]. Seasonal succession in bacterioplankton community composition is commonly driven by seasonal changes in environmental conditions such as water temperature, nutrients, and phytoplankton (as reviewed in [9]). On the other hand, seasonal variability of environmental conditions may depend on geographic factors such as latitude and distance to shore of the sampling stations [10,11]. With the change of latitude or distance to shore, multiple environmental gradients could be formed in coastal waters, thus leading to spatial turnover of bacterial communities [12,13]. Some previous studies have considered the coastal areas with ecological gradients as ideal models for understanding the interplay of seasonal and spatial variability in bacterioplankton communities [10,14]. However, the seasonality in biogeographic patterns and underlying processes of bacterioplankton along coastal environmental gradients is largely unknown.

The balance of seasonal and spatial variability of coastal bacterioplankton communities largely depends on spatial scale, environmental gradient strength, and their interactions. When at a local scale and/or across weak environmental gradients, seasonality in bacterioplankton community composition was likely more considerable compared with its spatial variability [15]. With an increase in spatial scale and/or environmental gradient strength, the larger changes of environmental conditions over space and/or enhanced dispersal limitation due to the longer geographic distance lead to a pattern where spatial variability overwhelms seasonality [14]. Spatial variability of bacterial communities in coastal waters can be simultaneously constrained by abiotic factors, including salinity [16,17], water temperature [7,18], suspended particles [13] and nutrients [7,19], and spatial factors [10,11] in various oceanic provinces. Biotic factors like phytoplankton biomass and composition can also be crucial drivers [20,21]. Collectively, spatial turnover of bacterial communities is likely governed by complex interactions among abiotic, biotic, and spatial factors. Therefore, a seasonal perspective on the relative influence of three categories of factors on shaping bacterial community composition is crucial to confirm whether the same factors would have similar explanatory power in spatial turnover of bacterial communities across seasons. 

Since the biogeographic patterns (beta-diversity patterns such as distance–decay relationship) of microbial communities and related factors have been extensively reported across global marine ecosystems [1,22,23,24], recent attention has been paid beyond the patterns to underlying processes [25,26]. The processes shaping biogeographic patterns involve two major categories: deterministic (mainly including heterogeneous selection and homogeneous selection) and stochastic (mainly including dispersal limitation, homogenizing dispersal, ecological drift due to random birth/death events, and diversification) [27]. The relative importance of selection and dispersal limitation on governing microbial community assembly in aquatic biomes is usually inferred by variation partitioning involving both environmental and spatial explanatory variables [13,23,28,29], but this aim often cannot be achieved when a large amount of variation explained by spatially structured environmental conditions and/or considerable variation remains unexplained, because measured/unmeasured environmental and spatial factors may both contribute to multiple fractions of compositional variation [27]. This is somewhat a pattern along coastal ecological gradients [13,30]. Some recent studies have employed null models based on both phylogenetic and taxonomic turnover [31] to quantify assembly processes of bacteria, archaea, and/or microeukaryotes in marine [26,32] and lacustrine waters [25,33]. However, the existence and extent of seasonality in processes governing microbial biogeography across ecological gradients are poorly estimated.

Many studies go beyond beta-diversity patterns to focus on specific taxonomic groups driving overall community variability across coastal environmental gradients [34,35,36]. For example, Fortunato et al. determined key bacterial taxa defining specific aquatic environments across a river-to-ocean gradient in the Columbia River coastal margin, and some of the indicator taxa also showed a seasonal signature [34]. Furthermore, seasonal switching between closely related bacterial taxa exhibiting distinct season preferences was found in the coastal marine waters, suggesting that seasonal succession of bacterial communities driven by switching between close relatives could be important for maintaining ecological functions in different seasons [35]. However, little is known about the seasonal consistency and specificity of the taxa driving the compositional variation of bacterial communities across the spatial gradient. Furthermore, phylogenetic relationships of these taxa in different seasons are unassessed.

**Figure 1:**
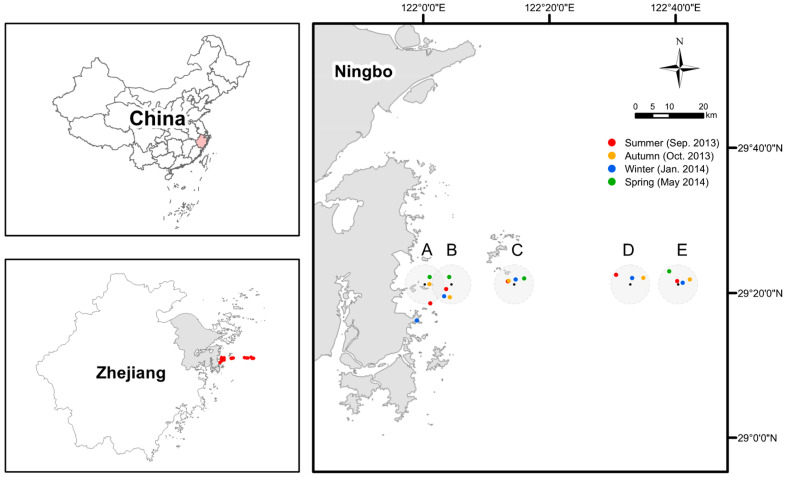
The map of sampling sites (with IDs as A, B, C, D, and E) and specific stations in each season. Due to the weather and sea conditions, the geographic coordinates of representative stations of each site actually being sampled are somewhat different in four seasons but within a radius of 5 km (except the one for site A in the winter, target site missed because of misreading the coordinates on board).

In this study, we sampled the surface waters along an ecological gradient from nearshore sites off Xiangshan coast to offshore sites over ~70 km in the East China Sea (Figure 1), as a representative system for studying spatio-seasonal variability of bacterial communities across surface waters with multiple environmental gradients [37]. Bacterioplankton communities were characterized by 16S rRNA gene sequencing data from four seasons. Using multivariate analyses and null models, we aim to answer three main questions: (1) Under the scenario that seasonal succession in bacterial community composition would likely occur, would bacterioplankton communities show seasonal variability in biogeographic patterns (e.g., distance–decay relationship or covariation of community composition and distance to shore) and underlying processes? (2) Would the same factors have a similar influence on driving spatial turnover of bacterioplankton across seasons? (3) What are the phylogenetic relationships and environmental preferences of the key taxa responsible for spatial turnover of bacterioplankton in different seasons?

## 2. Materials and Methods

### 2.1. Study Area, Sampling, and Analyses of Seawater Physicochemical Properties

We sampled surface waters (at the depth of ~0.5 m) from five sites along a nearshore-to-offshore gradient located off the eastern Xiangshan coast to the East China Sea in four cruises: late summer (Sep. 2013: transition between summer and autumn when the weather conditions were very close to mid-summer in the study area), autumn (Oct. 2013), winter (Jan. 2014), and spring (May 2014) (Figure 1). According to the water depth, sites A, B, and C are classified as nearshore sites, while sites D and E are classified as offshore sites [37]. Due to the weather and sea conditions, the geographic coordinates of representative stations of each site actually being sampled are somewhat different in four seasons but within a radius of 5 km (except the one for site A in the winter, where it was believed that the target site had been reached because of misreading the coordinates on board) (Figure 1). Five biological replicates were collected for each station in different seasons, except for site E (three replicates) in the late summer and site D (not sampled due to the terrible weather and sea conditions) in the spring. Thus, a total of 93 water samples were obtained. The water samples were pre-filtered through a sterilized 100-μm pore size nylon mesh, and then microbes were collected using a 0.2-μm pore size polycarbonate membrane (Millipore, Madison, WI, USA). The sterile tubes containing the filters were stored in a box with dry ice. The filters were brought back to the laboratory within 6 h and stored at −80 °C. Water temperature, pH, and dissolved oxygen (DO) were measured on board using a probe (YSI550A, Yellow Springs, OH, USA), while salinity was measured using a MASTER-S28M salinometer (ATAGO, Tokyo, Japan). Nitrate, ammonium, nitrite, total phosphorus (TP), phosphate, chemical oxygen demand (COD), and chlorophyll-*a* (Chl-*a*) were determined according to standard methods [38]. The concentration of dissolved inorganic nitrogen (DIN) is calculated as the sum of nitrate, ammonium, and nitrite. Total organic carbon (TOC) was determined using a multi N/C 3100 analyzer (Analytik Jena, Jena, Germany). The metadata including geographic coordinates of the sites and water environmental parameters were provided in Dataset S1.

### 2.2. DNA Extraction, 16S rRNA Gene Amplification, and Illumina Sequencing

Total DNA from the filters was extracted using PowerSoil DNA Isolation Kit (MOBIO, Jefferson City, MO, USA), which is suitable for water samples with a considerable amount of suspended particles. The V3-V4 region of bacterial 16S rRNA genes was amplified using primers 338F (5′-ACTCCTACGGGAGGCAGCAG-3′) and 806R (5′-GGACTACHVGGGTWTCTAAT-3′) with dual barcodes. An aliquot of 10 ng purified DNA template from each sample was amplified in triplicate with a 20-μL reaction system under the following conditions: denaturation at 95 °C for 3 min; then 28 cycles of denaturation at 95 °C for 30 s, annealing at 55 °C for 30 s, and extension at 72 °C for 45 s; with final extension at 72 °C for 10 min. Triplicated PCR products were pooled together, purified with magnetic beads, quantified using a Quant-It Pico Green kit with a Qubit fluorometer (Life Technologies, Carlsbad, CA, USA), and sequenced on an Illumina MiSeq machine (Illumina, San Diego, CA, USA). The sequence data are available under accession number PRJNA612952 in the BioProject of NCBI (https://www.ncbi.nlm.nih.gov/bioproject/).

### 2.3. Sequence Processing

The paired reads were joined using FLASH with default setting [39]. The joined pairs were then processed using QIIME 1.9.1 [40]. Briefly, the sequences were quality-controlled using the *split_libraries_fastq.py* script at Q20 [41]. The remaining sequences were chimera detected using UCHIME [42]. After filtering chimeras, the remaining sequences were clustered into operational taxonomic units (OTUs, >97% sequence similarity) using the *pick_open_reference_otus.py* script with the SortMeRNA & SUMACLUST method [43,44,45]. The most abundant sequence for a given OTU was selected as the representative sequence and then taxonomically assigned against SILVA 128 database. The representative sequence of OTUs were aligned using PyNAST [46], and a phylogenetic tree was generated from the filtered alignment using FastTree [47]. Archaea, chloroplast, mitochondria, and the sequences that were not assigned to bacteria were removed, as were singletons. A total of 2,169,998 clean bacterial reads, ranging from 12,457 to 32,318 per sample (mean 23,333) remained. To normalize the sequencing depth of each sample, the bacterial OTU table was rarefied at 12,450 sequences per sample using the QIIME script *single_rarefaction.py* for further analyses. We used chloroplast 16S rRNA gene data to present the composition of dominant eukaryotic phytoplankton [6]. A separate chloroplast OTU table was generated and then rarefied at 130 sequences per sample for further analyses. The representative sequences of chloroplast OTUs were taxonomically assigned against PhytoRef database [48] using Blastn [49] (the hit with the smallest *e*-value). 

### 2.4. General Statistical Analyses

We used ArcGIS 10.4 to calculate the nearest distance of each sampling station to the land (shore). Principal coordinate analysis (PCoA) was applied to visualize taxonomic (OTU) turnover based on Bray–Curtis dissimilarity using the QIIME script *beta_diversity_through_plots.py*. One-way and two-way crossed analysis of similarity (ANOSIM) were applied to test the significance of compositional difference of bacterial communities between sites across seasons using PRIMER-E v5.0 (PRIMER-E Ltd., Plymouth, UK). The distance-based multivariate linear model (DistLM) was applied to determine the key drivers of compositional variation of bacterial community using DISTLM forward 3 program [50]. Variation partitioning analysis (VPA) was applied to estimate the relative importance of abiotic, biotic, and spatial factors in shaping community composition with (partial-)Constrained analysis of principal coordinates (CAP) based on Bray–Curtis dissimilarity using the R package ‘vegan’ [51]. Eukaryotic phytoplankton taxa at the order level were involved as biotic variables. Spatial variables were derived from principal coordinates of neighbor matrices (PCNM) of geographic coordinates to obtain all detectable spatial scales [52]. Forward selection was performed to select the best subsets of abiotic, biotic, and PCNM variables, respectively, using DISTLM forward 3 program [50]. Significance tests were done with 999 permutations, and all R^2^ values were adjusted as described by Peres-Neto et al. [53]. Similarity percentage analysis (SIMPER) was applied to identify key OTUs dominantly responsible for spatial turnover of bacterial community composition in each season using PAST [54]. A heatmap, showing seasonal and spatial dynamics of the top 20 OTUs most contributing to the compositional variation of bacterial communities across the gradient in each season, was created by the R package ‘pheatmap’ [55]. A maximum-likelihood phylogenetic tree was constructed using MEGA 7 to present phylogenetic relationships among these OTUs [56].

### 2.5. Quantification of Bacterial Community Assembly Processes 

To use Stegen’s null modelling framework for inferring assembly processes of bacteria, it is a prerequisite to test phylogenetic signal in niche differences between species [31]. Briefly, the environmental optima for all bacterial OTUs were calculated as relative abundance-weighted mean values for the measured environmental variables, and niche differences between OTUs were calculated as Euclidean distances between optima for all variables [57]. We then used Mantel correlograms to estimate the correlation coefficients between niche differences and phylogenetic distances across different distance classes with the R function ‘mantel.correlog’ in the package ‘vegan’ [51]. Significance of these correlations was tested using 999 permutations with Holm correction.

We used a two-step null modelling approach to quantify the relative influence of the processes governing spatial turnover of bacterial communities [31]. Firstly, the deviation of observed phylogenetic turnover between communities from its null modelling distribution was measured to infer the balance between deterministic and stochastic assembly processes. If significant phylogenetic signal in between-OTU niche differences can be found across short phylogenetic distances, we can estimate phylogenetic turnover between communities by abundance-weighted β-mean nearest taxon distance (βMNTD) metric, calculated by the R function ‘comdistnt’ in the package ‘picante’ [31,58]. The distribution of null βMNTD (βMNTD_null_) was created by randomly shuffling OTUs on the phylogenetic tree 999 times. The difference between observed βMNTD (βMNTD_obs_) and the mean of βMNTD_null_ in the unit of standard deviations of the null distribution is expressed as SES.βMNTD (standardized effect size of βMNTD), also as known as β-nearest taxon index (βNTI). Significant deviation of βMNTD_obs_ from the null expectation with |βNTI| >2 indicates deterministic processes (selection). βNTI > +2 or < −2 infers heterogeneous selection or homogeneous selection in governing between-community differences or similarity, respectively. Nonsignificant βNTI values (|βNTI| < 2) infers stochastic processes in governing turnover between communities. The second step uses modified Raup-Crick metric [59] that evaluates the standardized deviation of observed Bray–Curtis dissimilarity from the null distribution (RC_bray_) to disentangle various stochastic processes [31]. The distribution of null Bray–Curtis dissimilarity was created by randomly assembling each pair of communities 999 times. When |βNTI| < 2, RC_bray_ > +0.95 or < −0.95 infers that dispersal limitation or homogenizing dispersal governs compositional difference or similarity between communities, respectively, while |RC_bray_| < 0.95 infers that compositional turnover between communities is undominated by any single process above and may be governed by multiple stochastic processes including drift, weak selection, and/or weak dispersal [27,60]. Finally, the percentages of each process among all pairwise comparisons were calculated according to the βNTI and RC_bray_ values.

## 3. Results

### 3.1. Spatial and Seasonal Changes of Abiotic Factors and Eukaryotic Phytoplankton

Nutrient-related factors including DIN, TP, and phosphate showed overall high to low gradients from nearshore to offshore sites in all seasons but lacked consistency in seasonal patterns across sites, except that the concentration of phosphate in all sites was significantly lower in spring compared with that in other seasons (Figure S1). Water temperature fit the common seasonal pattern in the subtropical area (summer > autumn ≈ spring > winter) and was stable among sites, while DO showed an overall opposite seasonal pattern. Salinity gradually increased from nearshore to offshore sites (except in the autumn) and showed somewhat seasonality. In addition, TOC showed a large spatial fluctuation without a unified seasonal pattern. The concentration of Chl-*a* in the summer and spring was overall higher than that in the autumn and winter. Since the relative abundance of cyanobacterial reads was much lower than that of chloroplast reads across space in all seasons (data not shown), eukaryotic phytoplankton likely contributed to most of the phytoplankton biomass in this area, corresponding to our previous report [37]. Eukaryotic phytoplankton communities (profiling based on the chloroplast 16S rRNA gene data) were dominated by Cryptophyta (41.5% in average across sites over seasons), Bacillariophyta (38.9%), Chlorophyta (4.9%), Raphidophyceae (4.8%), Haptophyta (2.4%), and Euglenozoa (1.2%) (Figure S2). In addition, clear seasonal succession was found in the composition of eukaryotic phytoplankton, which was dominated by Thalassiosirales, Bacillariales, and Pyrenomonadales in the summer; Thalassiosirales, Cymatosirales, and Pyrenomonadales in the autumn; Thalassiosirales and Pyrenomonadales in the winter and spring (Figure S2).

### 3.2. Seasonal Patterns of Dominant Bacterial Taxa along the Gradient

**Figure 2:**
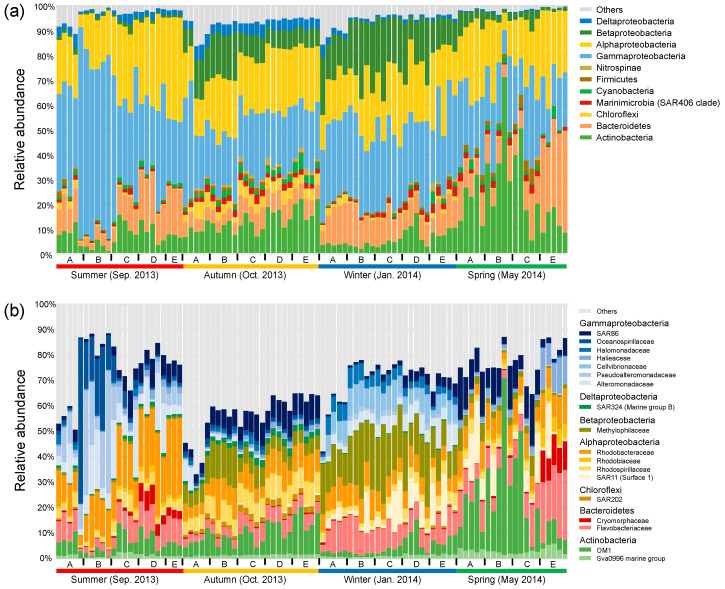
Relative abundance of dominant bacterial phyla and proteobacterial classes (average relative abundance >1% at least in one season) (**a**) and families (average relative abundance >2% at least in one season) (**b**).

In general, bacterial communities were predominated by Gammaproteobacteria (31.6% in average), Alphaproteobacteria (23.0%), Actinobacteria (12.7%), Bacteroidetes (11.6%), and Betaproteobacteria (9.2%) (Figure 2a). Seasonality in the relative abundance of dominant phyla or proteobacterial classes was overall stronger compared with their spatial shifts across sites. This overwhelming seasonal pattern was reflected by the highest abundance of Gammaproteobacteria (44.6%) in the summer, the highest abundance of Deltaproteobacteria (4.3%) and the balanced abundances of Alphaproteobacteria, Gammaproteobacteria, and Actinobacteria in the autumn, the highest abundance of Betaproteobacteria (18.7%) in the winter, and the highest abundance of Actinobacteria (24.7%) in the spring. The overwhelming seasonal pattern was also reflected at the family level, though the spatial variability was considerable, especially between nearshore and offshore sites in the summer and spring (Figure 2b). We observed seasonal transitions between families within the same phylum or proteobacterial class. Taking Gammaproteobacteria for example, Oceanospirillaceae, Pseudoalteromonadaceae, and Alteromonadaceae were overall enriched in the summer, SAR86 commonly peaked across the spatial gradient in the autumn and in the nearshore sites in the spring, and Halomonadaceae and Cellvibrionaceae were discriminatorily enriched in the winter. The class Alphaproteobacteria was overall dominated by Rhodobacteraceae in the summer, and by the balanced combination of Rhodobacteraceae and Rhodospirillaceae in the autumn, while the Suface_1 clade of SAR11 was enriched in the winter and spring.

### 3.3. Seasonal and Spatial Turnover of Bacterial Community Composition

**Figure 3:**
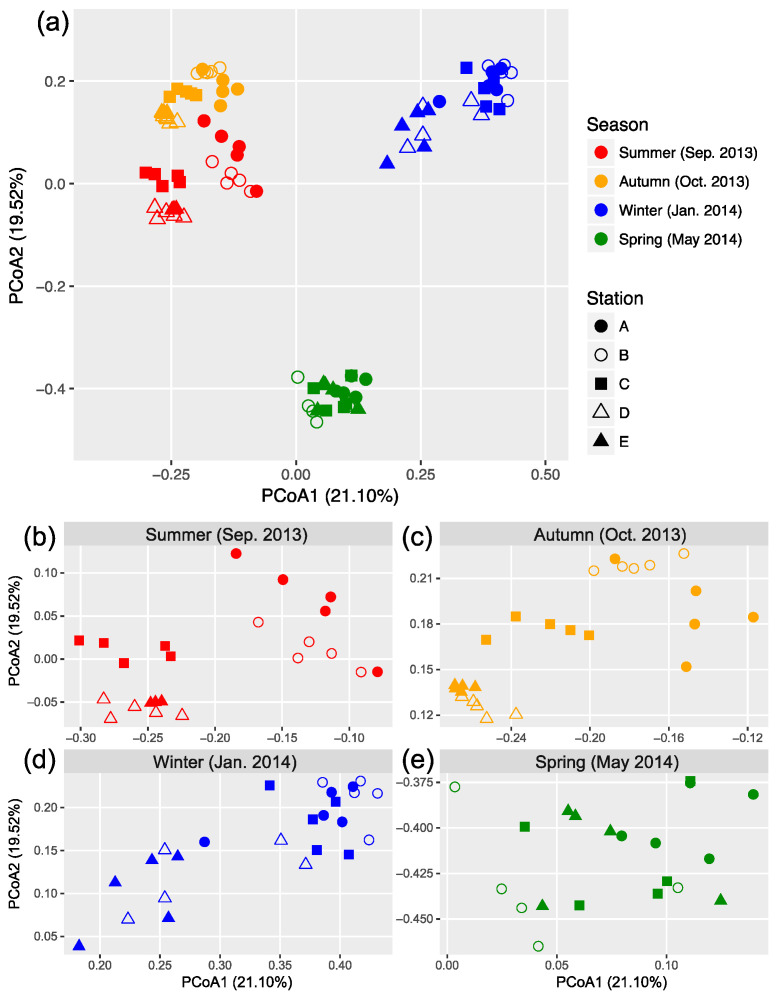
Principal coordinate analysis (PCoA) based on Bray–Curtis dissimilarity illustrating the variation in bacterial community composition across sites (**a**). Data from each season were expanded into separated panels to better illustrate the clustering patterns (**b**–**e**).

**Figure 4:**
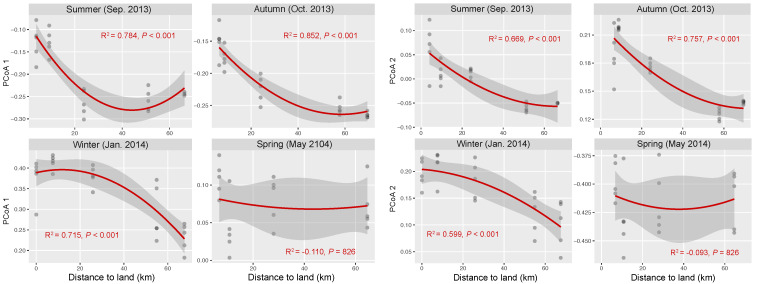
Correlations between bacterial community composition (indicated by the first two coordinates of PCoA) and the nearest distance to land of the stations in each season. The red lines present binomial fitting.

The OTU composition of bacterial communities varied with season (Figure 3a). The compositional difference between each pair of seasons or sites was generally significant as tested by two-way crossed ANOSIM (all *p* < 0.01), and the seasonality was stronger than spatial variability (Table S1). However, according to a finer view in each season, we did not find significant differences between two offshore sites (D and E) in the summer and winter and between sites B and C in the winter (Figure 3b–d and Table S2). In addition, there was no significant difference among the three nearshore sites in the spring (Figure 3e and Table S2). We found significant strong correlations between the first two PCoA coordinates (as indicators of community dissimilarity) and the nearest distance to land (DTL) of the sites in the summer, autumn, and winter (all *p* < 0.001), suggesting a gradual compositional turnover of bacterial community along the coastal gradient, but this was not the case in the spring (Figure 4). In addition, bacterial communities fit the distance–decay pattern in all seasons with overall more negative slope values in the winter and spring than those in the summer and autumn (Figure S3).

### 3.4. Drivers of Spatial Turnover of Bacterial Community Composition across Seasons

**Figure 5:**
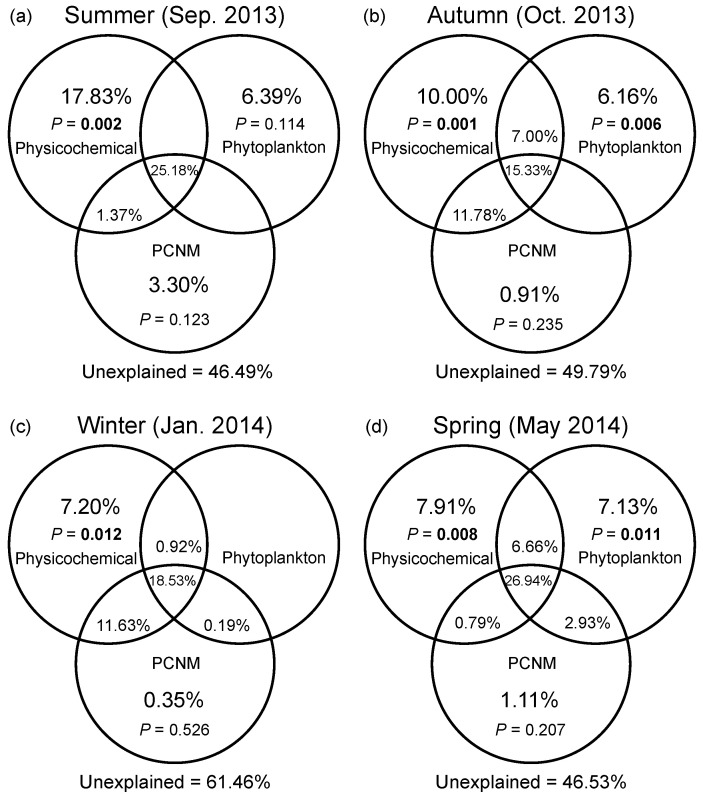
Variation partitioning of the Bray–Curtis dissimilarity between bacterial communities with physicochemical variables, eukaryotic phytoplankton, and spatial variables in the summer (**a**), autumn (**b**), winter (**c**), and spring (**d**). Physicochemical: physicochemical factors. Phytoplankton: eukaryotic phytoplankton at the order level based on the chloroplast 16S rRNA gene data. PCNM: spatial eigenvector converted from principal coordinates of neighbor matrices. Overlapped fractions represent the shared explained variance. Blank fractions present that R^2^ values < 0 after being adjusted.

DistLM based on a full-season dataset demonstrated that water temperature and DO were the most important environmental drivers of the seasonal variation in bacterial community composition (Table S3). Marginal tests of DistLM showed that nutrients (N- and P-related factors), salinity, and DTL all significantly contributed to spatial variation in bacterial community composition in four seasons. In general, VPA showed that the pure effect of spatial factors on compositional variation of bacterial communities was insignificant in all seasons (Figure 5a–d). Abiotic factors solely explained greater variation than eukaryotic phytoplankton solely did regardless of season, while the variation solely explained by phytoplankton reached the highest level in the spring (7.13%). The environmental factors (including abiotic and biotic factors) solely explained 24.2%, 23.2%, and 21.7% of variation in the summer, autumn, and spring, respectively; however, a pure environmental effect was weak in the winter. The shared fraction of environmental and spatial factors explained more variation than any category of factors solely did across seasons (ranging from 26.0% to 37.3%). In addition, the largest fraction of variation was still unexplained (ranging from 46.5% to 61.5%).

### 3.5. Processes Governing Bacterial Community Assembly

**Figure 6:**
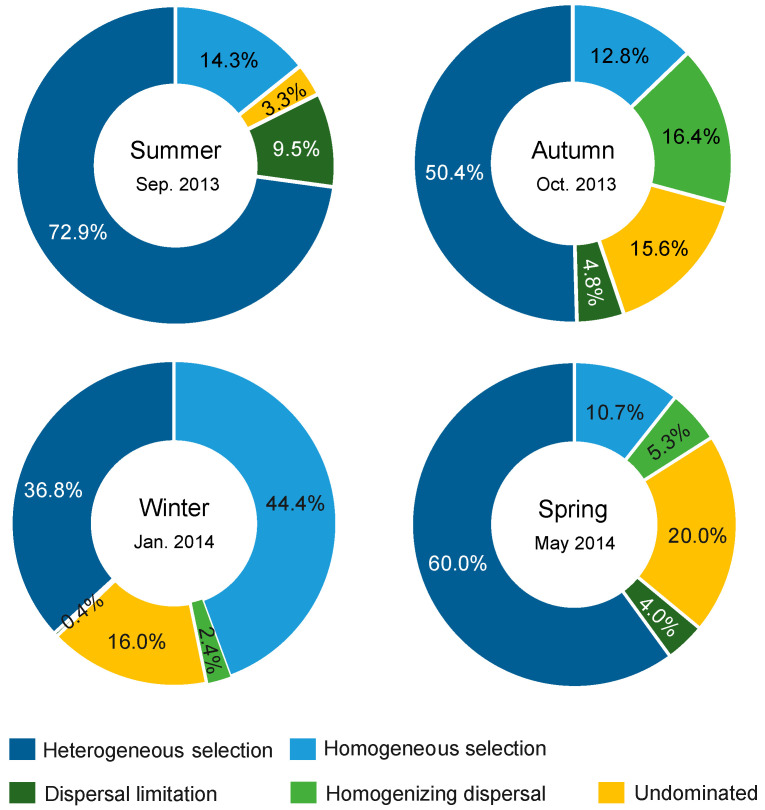
Summary of ecological processes governing bacterial community assembly in each season. The percentage of each process was the relative contribution to all pairwise comparisons between sites.

A significant phylogenetic signal was observed in the niche difference between bacterial OTUs across relatively short phylogenetic distances (Figure S4), fitting the assumption of phylogenetic null modelling based on βMNTD [31]. Bacterial community assembly was dominantly governed by deterministic processes relative to stochastic processes in four seasons (Figure 6). Selection governed 87.2%, 63.2%, 81.2%, and 70.7% of community turnover in the summer, autumn, winter, and spring, respectively. In all seasons, selection (mainly heterogeneous selection in the summer, autumn and spring, and a balanced combination of heterogeneous and homogeneous selections in the winter) was 8.7–17.8 times more important than dispersal-related processes in governing bacterial community assembly. In addition, a very low proportion of compositional turnover was undominated by any single process (probably by a combination of drift, weak selection, or/and weak dispersal [27]) in the summer, but these stochastic processes were considerable in the other three seasons (corresponding to 15.6–20.0% of community turnover).

### 3.6. Key Taxa Responsible for Spatial Turnover of Bacterioplankton in Different Seasons

**Figure 7:**
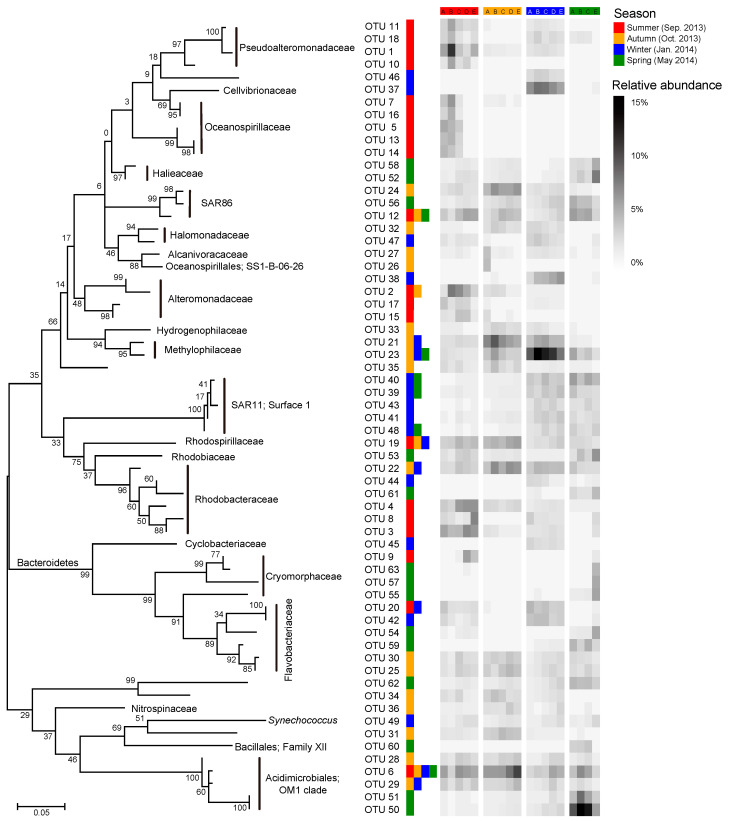
Dynamics of key bacterial OTUs (operational taxonomic units) dominantly responsible for spatial turnover of bacterial community composition across sites over seasons. The heatmap shows average relative abundance of the top 20 OTUs contributing the most compositional variation of bacterial communities along the gradient in each season as identified by Similarity percentage analysis (SIMPER; see detailed results of SIMPER in Dataset S2). The color keys following a given OTU indicate that the OTU was a key driver of spatial turnover in specific season(s). The phylogenetic tree was constructed using MEGA 7.0 with the maximum likelihood method.

**Figure 8:**
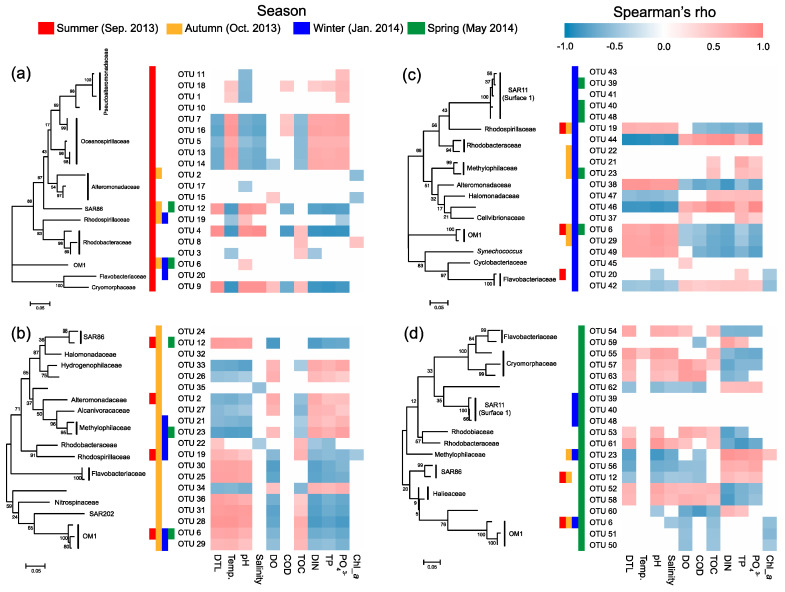
Spearman’s correlations between the top 20 key driver OTUs and environmental factors in the summer (**a**), autumn (**b**), winter (**c**), and spring (**d**). DIN, dissolved inorganic nitrogen; TP, total phosphorus; Temp., water temperature; COD, chemical oxygen demand; DO, dissolved oxygen; TOC, total organic carbon. Chl-*a*, chlorophyll-*a*. Colored cells in the heatmaps indicate significant correlations (*p* < 0.05).

Most of the key OTUs that dominantly drove the spatial turnover of bacterial communities showed a season-specific pattern, while 20.6% of the key driver OTUs showed some ubiquity in multiple seasons with only one in all seasons (Figure 7). The key driver OTUs in the summer were mainly from the families Pseudoalteromonadaceae, Oceanospirillaceae, Alteromonadaceae, and Rhodobacteraceae. Most Pseudoalteromonadaceae OTUs were positively correlated with phosphate, and negatively correlated with pH, while the Oceanospirillaceae OTUs showed consistency in their responding pattern to environmental conditions, that is, positive correlations with water temperature and N- and P-nutrients, and negative correlations with DTL, salinity, pH, and TOC (Figure 8a). However, the Rhodobacteraceae OTUs showed distinct responding patterns to environmental conditions, while the Alteromonadaceae OTUs showed a very weak association with measured environmental factors. The key driver assemblages were more phylogenetically diverse in the autumn, including members of SAR86, Halomonadaceae, Alcanivoracaceae, Alteromonadaceae, Hydrogenophilaceae, Methylophilaceae, Rhodospirillaceae, Rhodobacteraceae, Flavobacteriaceae, Nitrospinaceae, and OM1 (Figure 7). In general, these OTUs showed two opposite responding patterns to environmental conditions and some taxonomic dependency (Figure 8b). In the winter, we found a shift of driver assemblages to SAR11 (Surface_1), Rhodobacteraceae, Flavobacteriaceae, and OM1, and then to Halieaceae, SAR86, SAR11 (Surface_1), Cryomorphaceae, Flavobacteriaceae, and OM1 in the spring (Figure 7). Most of the key OTUs in both winter and spring were associated with environmental conditions, except that all the SAR11 OTUs were not significantly correlated with any measured environmental factors (Figure 8c,d).

We further found seasonal switching between closely related OTUs, responsible for spatial turnover of bacterial communities, within many families including SAR86, Alteromonadaceae, Rhodobacteraceae, Cryomorphaceae, Flavobacteriaceae, and OM1 (Figure 7). However, these close relatives commonly exhibited distinct patterns in their distribution from nearshore to offshore (Figure 7) as well as in response to environmental conditions (Figure 8) in different seasons. In addition, the key driver OTUs shared by multiple seasons also showed seasonal inconsistency in the distribution patterns along the gradient and the associations with environmental conditions (Figure 7 and Figure 8).

## 4. Discussion

### 4.1. Seasonal Succession Patterns of Bacterioplankton

We found that seasonality overwhelmed spatial variability of bacterial community composition. In contrast, at the similar spatial scale, the spatial variability dominated patterns were found across a river-to-ocean gradient in the Columbia River margin [14] and along a nearshore-to-offshore gradient in the Sargasso Sea [10], corresponding to the stronger environmental gradients over space (salinity in the former and water temperature in the latter) compared with those in this work. Here, the strong seasonal succession in bacterioplankton could be due to the strong seasonal changes in water temperature and DO concentration. The *Tara Ocean* project has demonstrated temperature and DO as two most influential factors shaping global patterns of marine bacterioplankton [1]. Moreover, seasonal shifts in bacterioplankton composition also commonly follow the changes in Chl-*a* in temperate/subtropical regions [4,7,9,61]. We also found that Chl-*a* played a role in driving bacterioplankton seasonal succession, as Chl-*a* only significantly contributed to the compositional variation of bacterioplankton when the all-season dataset was considered.

The seasonal succession patterns of bacterioplankton were also pronounced at the family level, as were differences between nearshore and offshore sites, especially in the summer and spring. For example, Flavobacteriaceae and Cryomorphaceae (Bacteroidetes) were only enriched in the offshore sites compared with nearshore sites in the summer and spring. Members of Bacteroidetes are versatile in degradation of phytoplankton-derived dissolved organic matter (DOM), and typically become dominant in bacterioplankton in productive seasons [62,63,64,65]. This suggests phytoplankton blooms and/or unique phytoplankton composition in the offshore waters in the summer and spring, which was evidenced by overall higher Chl-*a* concentration in both seasons and the unique composition of eukaryotic phytoplankton in the spring. In addition, Rhodobacteraceae with members frequently associated with DOM degradation during phytoplankton blooms [9,66] was also enriched in the offshore waters in the summer. The seasonal succession patterns largely depend on the distance to shore, which typically structures pronounced gradients in salinity and nutrients from coastal zones to open ocean [9]. Given the overall seasonal consistency in the gradients of salinity and N- and P-nutrients in the study area, the interplay between abiotic environmental conditions and phytoplankton dynamics likely determined the distinct seasonal succession patterns of bacterioplankton between nearshore and offshore waters, though temperature and DO shaped the major succession trend for the entire area.

### 4.2. Seasonality in Biogeographic Patterns of Bacterioplankton and Underlying Processes

Under the baseline of strong seasonal succession in bacterial composition, we also found overall significant spatial turnover of bacterial communities. Many previous studies have characterized the spatial turnover of bacterioplankton composition along coastal gradients [13,67,68,69], but the extent of seasonality in biogeographic patterns and the underlying processes are largely unknown. In this study, we tested two biogeographic patterns: (i) covariation of community composition and distance to shore (land) (Pattern I) and (ii) distance–decay relationship (Pattern II). Wang et al. found significant Pattern I in the Sargasso Sea by integrating data from many different months for years [10], but the existence of seasonality was not concerned. In our work, seasonal consistency in Pattern I was evidenced by significant correlation between DTL and PCoA 1 and 2 coordinates in the summer, autumn, and winter, but the spatial turnover of bacterial communities in the spring did not fit Pattern I. On the other hand, compositional variation of bacterial communities fit Pattern II, but with variability in spatial turnover rate as indicated by the slope of the model. This suggests the co-existence of certain seasonal consistency and subtle dynamics in biogeographic patterns of bacterioplankton along the coastal gradient.

Null modelling revealed a consistent determinism (selection)-overwhelming mechanism governing bacterial community assembly across seasons. Using the same approach, Wu et al. found that selection was 1.4 times more important than dispersal limitation in shaping bacterial communities in the open ocean of East China Sea [26], while Logares et al. found that prokaryotic communities were governed, in a balanced manner, by selection, dispersal limitation, and drift in global sunlit-oceans [70]. The more selection-overwhelming pattern in the present area relative to these two surveys could be due to the smaller scale of ours, at which dispersal limitation is expected to be weak [71]. Pattern II can be driven by selection, dispersal limitation, and/or drift when interplayed with dispersal limitation [23]. The overall weak dispersal limitation in all seasons was corresponding to the insignificant pure effect of spatial factors in VPA, suggesting that the consistent distance–decay pattern here should be mainly governed by selection. Unlike the heterogeneous selection-dominance in the summer, autumn, and spring, bacterial community assembly in the winter was governed by both heterogeneous and homogeneous selections in balance, which is corresponding to the less compositional variation explained by variation (heterogeneity) in environmental conditions (including pure and shared effects in the VPA) than that in the other three seasons. On the other hand, the relative importance of stochastic assembly processes of bacterioplankton showed seasonal shifts with higher stochasticity in the autumn and spring. The mediators of the balance of deterministic and stochastic assembly processes of bacterioplankton in dynamic marine ecosystems should be considered as a future direction. Collectively, to some extent, we found consistency in a determinism-dominated mechanism underlying bacterial community assembly across seasons as well as the existence of seasonality in actual processes.

### 4.3. Relative Importance of the Factors Driving Spatial Turnover of Bacterioplankton

Many studies have considered salinity as the master factor shaping bacterial community composition across coastal gradients from estuary to ocean [14,16,72]. We also found salinity associated with compositional variation of bacterioplankton along the gradient in three seasons (except autumn). A recent study found that bacterial communities across a nearshore-to-offshore gradient in the Sargasso Sea were primarily shaped by distance to shore and temperature [10]. Water temperature and DTL were also potential drivers of compositional variation of bacterial in the summer, autumn, and winter as revealed by DistLM (Table S3). Moreover, N- and P-related factors showed seasonal similarity in the extent they influenced the compositional variation of bacterial communities along the spatial gradient, corresponding to their overall high to low gradients from nearshore to offshore in four seasons. To some extent, these results suggest seasonal consistency in abiotic factors shaping bacterial community composition. 

Some recent works have provided evidence about the greater importance of biotic interactions relative to biotic environmental conditions in coastal waters during certain periods like spring phytoplankton bloom [6]. However, the biotic factors represented by phytoplankton were overall less important in driving spatial turnover of bacterioplankton, as indicated by the insignificant effect of Chl-*a* (as an indicator of phytoplankton biomass) on compositional variation of bacterioplankton in each season (Table S3). Thus, phytoplankton could only act as a driver of seasonal succession of bacterioplankton, since the effect of Chl-*a* on compositional variation was only significant across seasons. The insignificant or weak pure effect of eukaryotic phytoplankton taxa in the VPA of summer, autumn, and winter further confirmed that not only biomass but also composition of phytoplankton played less important roles in spatial turnover of bacterioplankton. Only in the spring eukaryotic phytoplankton showed a similar influence as abiotic factors in shaping bacterial communities along the spatial gradient, corresponding to the largest between-site variation in phytoplankton composition in this season.

Since many abiotic factors (including water temperature, pH, DIN, TP, phosphate) were auto-correlated with DTL (Table S4), the influence of two categories of factors may not be detangled. The VPA also demonstrated that a large amount of variation was explained by a shared fraction of environmental and spatial factors. However, the overwhelming role of selection in governing spatial turnover of bacterial communities suggests that environmental factors (varied with DTL) mainly drove the covariation pattern of community composition and DTL (Pattern I). In addition, the largest community variation remaining unexplained did not necessarily mean high stochasticity in bacterial community assembly but indicated the existence of unmeasured environmental drivers of selection in our survey. Collectively, the quantification of community assembly processes provides a powerful supplement to distinguish the relative importance of environmental and spatial factors in shaping bacterial communities.

### 4.4. Phylogenetic Perspective and Environmental Preference of the Key Taxa Responsible for Spatial Turnover of Bacterioplankton

To understand how individual taxa as internal drivers of bacterioplankton spatial turnover rather than the whole community respond to environmental conditions (as external drivers), we identified the key driver OTUs responsible for a large amount of compositional variation of bacterioplankton in each season. Although some key driver OTUs, such as the members of Pseudoalteromonadaceae and Oceanospirillaceae, showed season-specificity, we found seasonal switching between closely related OTUs within most dominant bacterial families. Switching between closely related species is a common process in seasonal succession of microbial communities in the temperate/subtropical sites [35,73]. For example, switching between phylogenetically close OTUs with either summer or winter preferences was observed within the families Rhodobacteraceae, Synechococaceae, and Cryomorphaceae in the Pivers Island Coastal Observatory (PICO) site and this process was repeated annually [35]. Distinct Thaumarchaeota Marine Group I and *Nitrospina* assemblages were likely partners responsible for complete nitrification in the SPOT station at different times of the year [74]. Ecological divergence in closely related microbes was typically found along spatial and temporal gradients [75], which has been considered as a strategy for microbial communities to maintain specific ecological functions under dynamic conditions over space and time. Beyond the perspective from a single time-series site, we further revealed that many close relatives showed different responding patterns to the environmental gradients in different seasons, suggesting their differences not only in seasonally climatic preference but also in spatially environmental preference. Our results somewhat reflect the complex interplay of time and space in diversification of bacterioplankton. On the other hand, we also found taxonomic and phylogenetic dependency in environmental responding patterns in each season (especially in the autumn); that is, the driver OTUs from the same bacterial family or being more phylogenetically close likely respond to the environmental conditions in more similar manners in the same season. Therefore, the distinct distribution patterns of closely related OTUs along the gradients in different seasons could also be due to the interactions of unmeasured environmental factors (such as DOM composition) with those that have been characterized in this study. These interactions may also partly explain the seasonal inconsistency in the distribution pattern and the associations with the same environmental factors of many key driver OTUs shared by multiple seasons. Another explanation is that these shared OTUs showed less preference of specific season, which may hold wider niche breadth, thus enhancing the stochasticity in spatial distribution and environmental preference.

## 5. Conclusions

This study confirms the existence of seasonality in spatial turnover of coastal bacterioplankton along a nearshore-to-offshore gradient in the East China Sea. Our results go beyond the seasonal succession patterns in bacterioplankton in composition to the seasonal consistency and variability in their spatial turnover by disentangling the biogeographic patterns, ecological processes, and external and internal drivers across seasons, highlighting the importance of a seasonal perspective on bacterial community assembly in marine ecosystems. Furthermore, we found similar or distinct environmental preferences of the closely related taxa responsible for bacterioplankton spatial turnover in the same or different season(s), respectively, indicating the complex interplay of time and space in diversification of marine bacterioplankton.

## Supplementary Materials

The following are available online at https://www.mdpi.com/2076-2607/8/10/1484/s1, Figure S1: Spatiotemporal dynamics of water environmental parameters across five sites over seasons. Figure S2: Relative abundance of dominant eukaryotic phytoplankton taxa (upper) and principal coordinate analysis (PCoA) based on Bray-Curtis dissimilarity illustrating the variations in eukaryotic phytoplankton community composition (bottom) based on the Chloroplast 16S rRNA gene data across sites over seasons. Figure S3: Significant correlations between Bray-Curtis similarity and geographic distance showing the distance-decay pattern in the similarity between bacterial communities. Figure S4: Mantel correlograms testing phylogenetic signal in ecological niches of bacterial OTUs across between-OTU phylogenetic distance classes in four seasons. Table S1: Two-way crossed analysis of similarity (ANOSIM) of bacterial community composition based on Bray-Curtis dissimilarity across seasons and sampling sites (999 permutations). Table S2: One-way analysis of similarity (ANOSIM) of bacteria community composition based on Bray-Curtis dissimilarity between sites in each season (999 permutations). Table S3: Distance-based multivariate linear model (DistLM) of compositional variation of bacterial community (based on Bray-Curtis dissimilarity) against environmental variables with 999 permutations. Table S4: Spearman’s correlation coefficients between geo-environmental factors. Dataset S1: Geo-environmental variables of the sampling sites in this study. Dataset S2: Similarity percentage analysis (SIMPER) showing key driver OTUs responsible for spatial turnover of bacterioplankton in each season.
