# Supplementary material for: Seasonality in Spatial Turnover of Bacterioplankton Along an Ecological Gradient in the East China Sea: Biogeographic Patterns, Processes and Drivers
Source: Microorganisms. 2020 Sep 27;8(10):1484. doi: 10.3390/microorganisms8101484 (PMC7600760; doi:10.3390/microorganisms8101484)
Supplement: Supplementary file 1 [file microorganisms-08-01484-s001.zip › Supplementary Dataset S1.pdf]

Dataset S1 Geo-environmental variables of the sampling sites in this study.

Sample ID	Longitude (E)	Latitude (N)	Season	Site	water temperture (°C)	pH	Salinity (‰)	DO (mg/L)	COD (mg/L)	TOC (mg/L)	DIN (mg/L)	TP (mg/L)	Phosphate-P (mg/L)	Chl-*a* (μg/L)
13091501	122.0186	29.3098	Summer	A	25.0	8.10	25.98	6.91	0.95	1.43	0.524	0.1543	0.0396	10.0
13091502	122.0186	29.3098	Summer	A	25.0	8.07	25.95	7.02	1.00	1.44	0.545	0.1524	0.0401	26.2
13091503	122.0186	29.3098	Summer	A	25.1	8.07	26.04	6.94	0.91	1.44	0.522	0.1321	0.0387	30.8
13091504	122.0186	29.3098	Summer	A	25.1	8.09	26.12	7.07	0.97	1.46	0.540	0.1493	0.0410	13.8
13091505	122.0186	29.3098	Summer	A	25.1	8.10	26.03	6.95	0.94	1.45	0.550	0.1501	0.0391	20.7
13091601	122.0610	29.3426	Summer	B	25.1	8.11	26.35	7.10	0.84	1.64	0.493	0.0824	0.0409	4.7
13091602	122.0610	29.3426	Summer	B	25.0	8.09	26.41	7.07	0.81	1.61	0.485	0.0742	0.0389	4.4
13091603	122.0610	29.3426	Summer	B	25.1	8.10	26.33	6.87	0.77	1.64	0.479	0.0731	0.0377	3.5
13091604	122.0610	29.3426	Summer	B	25.2	8.08	26.28	6.93	0.90	1.59	0.497	0.0882	0.0401	38.2
13091605	122.0610	29.3426	Summer	B	25.2	8.09	26.37	6.88	0.95	1.60	0.495	0.0784	0.0411	24.7
13091801	122.2241	29.3606	Summer	C	24.9	8.11	27.11	7.12	0.74	1.45	0.427	0.0656	0.0371	5.7
13091802	122.2241	29.3606	Summer	C	24.9	8.12	27.19	7.04	0.70	1.47	0.432	0.0658	0.0367	3.8
13091803	122.2241	29.3606	Summer	C	24.9	8.13	27.21	7.06	0.79	1.41	0.435	0.0623	0.0345	3.3
13091804	122.2241	29.3606	Summer	C	25.0	8.13	27.09	6.94	0.68	1.43	0.436	0.0622	0.0353	8.2
13091805	122.2241	29.3606	Summer	C	25.0	8.12	27.15	6.97	0.73	1.42	0.437	0.0636	0.0348	4.5
13092001	122.5095	29.3756	Summer	D	24.8	8.13	28.35	7.20	0.80	1.67	0.387	0.0504	0.0329	6.2
13092002	122.5095	29.3756	Summer	D	24.7	8.14	28.23	7.16	0.73	1.66	0.379	0.0483	0.0337	7.0
13092003	122.5095	29.3756	Summer	D	24.8	8.14	28.41	7.07	0.69	1.65	0.392	0.0499	0.0332	10.4
13092004	122.5095	29.3756	Summer	D	24.8	8.12	28.36	7.24	0.74	1.67	0.369	0.0482	0.0323	18.8
13092005	122.5095	29.3756	Summer	D	24.7	8.12	28.33	7.15	0.85	1.67	0.374	0.0512	0.0354	5.4
13092101	122.6709	29.3611	Summer	E	24.7	8.14	29.23	7.00	0.70	1.73	0.359	0.0358	0.0321	12.7
13092102	122.6709	29.3611	Summer	E	24.7	8.14	29.21	6.98	0.65	1.71	0.364	0.0348	0.0332	33.1
13092103	122.6709	29.3611	Summer	E	24.7	8.13	29.18	6.84	0.74	1.72	0.376	0.0355	0.0327	10.1
13101501	122.0168	29.3538	Autumn	A	20.4	8.05	25.91	8.31	0.65	1.59	0.564	0.1239	0.0389	5.8
13101502	122.0168	29.3538	Autumn	A	20.5	8.06	25.88	8.26	0.66	1.57	0.554	0.1462	0.0397	2.9
13101503	122.0168	29.3538	Autumn	A	20.5	8.05	25.81	8.09	0.63	1.58	0.550	0.1201	0.0412	5.9
13101504	122.0168	29.3538	Autumn	A	20.4	8.04	25.79	7.95	0.80	1.59	0.563	0.1467	0.0401	2.8
13101505	122.0168	29.3538	Autumn	A	20.5	8.05	25.77	7.97	0.76	1.58	0.568	0.1436	0.0397	6.1
13101601	122.0705	29.3238	Autumn	B	20.6	8.05	25.71	7.88	0.59	1.64	0.513	0.0586	0.0399	4.1
13101602	122.0705	29.3238	Autumn	B	20.5	8.06	25.73	7.96	0.68	1.62	0.528	0.0623	0.0389	3.7
13101603	122.0705	29.3238	Autumn	B	20.6	8.05	25.74	7.95	0.69	1.64	0.517	0.0712	0.0384	3.4
13101604	122.0705	29.3238	Autumn	B	20.5	8.06	25.73	7.85	0.84	1.63	0.514	0.0687	0.0392	4.5
13101605	122.0705	29.3238	Autumn	B	20.6	8.06	25.69	7.96	0.75	1.61	0.514	0.0647	0.0402	4.4
13101801	122.2258	29.3614	Autumn	C	20.8	8.09	26.43	7.89	0.79	1.45	0.458	0.0654	0.0385	2.2
13101802	122.2258	29.3614	Autumn	C	20.8	8.08	26.45	7.82	0.77	1.43	0.444	0.0657	0.0389	2.9

13101803	122.2258	29.3614	Autumn	C	20.7	8.10	26.43	7.94	0.69	1.42	0.448	0.0595	0.0366	5.6
13101804	122.2258	29.3614	Autumn	C	20.9	8.10	26.44	7.89	0.64	1.43	0.455	0.0616	0.0371	5.1
13101805	122.2258	29.3614	Autumn	C	20.7	8.09	26.45	7.72	0.67	1.41	0.437	0.0627	0.0359	5.1
13102001	122.5814	29.3686	Autumn	D	21.6	8.10	26.13	7.95	0.72	1.67	0.387	0.0412	0.0318	4.2
13102002	122.5814	29.3686	Autumn	D	21.5	8.11	26.11	7.72	0.63	1.66	0.378	0.0402	0.0323	4.4
13102003	122.5814	29.3686	Autumn	D	21.6	8.11	26.07	7.63	0.61	1.65	0.375	0.0416	0.0319	9.1
13102004	122.5814	29.3686	Autumn	D	21.5	8.10	26.11	7.83	0.67	1.66	0.383	0.0409	0.0322	8.4
13102005	122.5814	29.3686	Autumn	D	21.6	8.12	26.09	7.68	0.65	1.65	0.378	0.0432	0.0331	8.9
13102101	122.7043	29.3647	Autumn	E	21.8	8.11	25.33	7.60	0.85	1.69	0.338	0.0338	0.0286	2.2
13102102	122.7043	29.3647	Autumn	E	21.7	8.12	25.33	7.76	0.77	1.71	0.347	0.0348	0.0279	2.7
13102103	122.7043	29.3647	Autumn	E	21.8	8.11	25.32	7.71	0.71	1.72	0.333	0.0338	0.0295	1.2
13102104	122.7043	29.3647	Autumn	E	21.8	8.12	25.33	7.68	0.72	1.74	0.336	0.0351	0.0288	1.6
13102105	122.7043	29.3647	Autumn	E	21.9	8.12	25.32	7.76	0.66	1.75	0.338	0.0341	0.0269	1.3
14011501	121.9835	29.2705	Winter	A	10.1	8.10	26.08	9.89	0.85	1.67	0.738	0.0406	0.0376	1.2
14011502	121.9835	29.2705	Winter	A	10.0	8.11	26.77	9.92	0.83	1.68	0.722	0.0418	0.0373	0.7
14011503	121.9835	29.2705	Winter	A	10.1	8.10	26.84	10.15	0.80	1.66	0.722	0.0422	0.0370	0.8
14011504	121.9835	29.2705	Winter	A	10.0	8.11	26.64	9.94	0.78	1.63	0.697	0.0413	0.0370	0.8
14011505	121.9835	29.2705	Winter	A	10.0	8.11	26.85	9.86	0.77	1.62	0.695	0.0429	0.0367	1.1
14011601	122.0556	29.3259	Winter	B	10.2	8.11	26.72	9.97	0.81	1.76	0.630	0.0443	0.0373	0.8
14011602	122.0556	29.3259	Winter	B	10.2	8.11	27.11	10.29	0.77	1.73	0.634	0.0451	0.0391	0.7
14011603	122.0556	29.3259	Winter	B	10.1	8.11	27.21	9.73	0.75	1.73	0.580	0.0426	0.0385	0.6
14011604	122.0556	29.3259	Winter	B	10.2	8.12	27.07	10.32	0.71	1.71	0.597	0.0419	0.0367	0.8
14011605	122.0556	29.3259	Winter	B	10.2	8.12	27.13	10.08	0.85	1.69	0.609	0.0435	0.0388	0.6
14011801	122.2444	29.3648	Winter	C	10.9	8.14	27.94	9.93	0.76	1.53	0.530	0.0495	0.0351	1.2
14011802	122.2444	29.3648	Winter	C	11.0	8.15	27.98	9.74	0.64	1.51	0.530	0.0485	0.0348	0.8
14011803	122.2444	29.3648	Winter	C	11.0	8.15	28.12	10.27	0.83	1.49	0.472	0.0488	0.0351	0.7
14011804	122.2444	29.3648	Winter	C	11.0	8.15	28.15	10.09	0.68	1.48	0.466	0.0467	0.0333	1.0
14011805	122.2444	29.3648	Winter	C	10.9	8.15	28.18	9.54	0.77	1.46	0.512	0.0473	0.0339	1.3
14012001	122.5521	29.3681	Winter	D	11.8	8.19	29.97	9.12	0.45	1.49	0.325	0.0305	0.0237	1.6
14012002	122.5521	29.3681	Winter	D	11.9	8.20	30.05	8.97	0.54	1.47	0.310	0.0319	0.0246	1.2
14012003	122.5521	29.3681	Winter	D	11.8	8.20	30.16	10.00	0.52	1.45	0.338	0.0333	0.0246	1.5
14012004	122.5521	29.3681	Winter	D	11.8	8.19	30.06	9.53	0.54	1.39	0.328	0.0321	0.0225	1.3
14012005	122.5521	29.3681	Winter	D	11.9	8.19	30.09	9.48	0.45	1.41	0.322	0.0316	0.0225	1.1
14012101	122.6855	29.3571	Winter	E	12.4	8.19	31.39	9.83	0.53	1.39	0.275	0.0221	0.0169	0.8
14012102	122.6855	29.3571	Winter	E	12.5	8.19	31.52	9.16	0.46	1.37	0.260	0.0226	0.0169	0.6
14012103	122.6855	29.3571	Winter	E	12.5	8.20	31.53	9.41	0.51	1.38	0.224	0.0228	0.0166	1.2
14012104	122.6855	29.3571	Winter	E	12.5	8.20	31.67	9.35	0.52	1.37	0.240	0.0223	0.0184	0.6
14012105	122.6855	29.3571	Winter	E	12.5	8.19	31.57	9.02	0.53	1.35	0.216	0.0215	0.0191	1.5
14051501	122.0173	29.3701	Spring	A	20.8	8.10	27.44	8.05	0.97	1.61	0.644	0.0702	0.0263	9.4

14051502	122.0173	29.3701	Spring	A	20.8	8.10	27.61	7.96	0.87	1.63	0.622	0.0691	0.0257	11.3
14051503	122.0173	29.3701	Spring	A	20.9	8.09	27.95	7.97	1.05	1.61	0.617	0.0648	0.0239	8.6
14051504	122.0173	29.3701	Spring	A	20.8	8.09	27.91	7.97	1.01	1.58	0.649	0.0712	0.0251	8.5
14051505	122.0173	29.3701	Spring	A	20.9	8.10	27.81	8.02	0.75	1.57	0.632	0.0566	0.0259	9.2
14051601	122.0687	29.3701	Spring	B	20.9	8.12	27.94	8.05	0.68	1.55	0.519	0.0536	0.0233	6.5
14051602	122.0687	29.3701	Spring	B	20.8	8.11	28.29	7.96	0.83	1.62	0.516	0.0499	0.0241	6.9
14051603	122.0687	29.3701	Spring	B	20.9	8.13	28.22	6.48	0.91	1.62	0.527	0.0497	0.0231	4.5
14051604	122.0687	29.3701	Spring	B	20.9	8.11	28.15	7.95	1.12	1.55	0.517	0.0512	0.0251	7.7
14051605	122.0687	29.3701	Spring	B	20.9	8.12	28.16	7.94	0.73	1.62	0.518	0.0501	0.0239	7.1
14051801	122.2669	29.3670	Spring	C	21.1	8.12	28.53	7.74	0.76	1.57	0.536	0.0494	0.0225	4.2
14051802	122.2669	29.3670	Spring	C	21.2	8.13	28.71	7.88	0.72	1.59	0.540	0.0497	0.0228	4.8
14051803	122.2669	29.3670	Spring	C	21.1	8.12	28.74	7.84	0.82	1.56	0.540	0.0501	0.0231	7.3
14051804	122.2669	29.3670	Spring	C	21.1	8.11	28.89	7.76	0.79	1.62	0.539	0.0534	0.0231	13.2
14051805	122.2669	29.3670	Spring	C	21.2	8.12	28.65	7.88	0.72	1.55	0.546	0.0552	0.0223	7.7
14052101	122.6501	29.3834	Spring	E	21.0	8.52	30.75	8.87	1.62	1.63	0.111	0.0121	0.0041	7.8
14052102	122.6501	29.3834	Spring	E	21.0	8.49	30.75	8.79	1.56	1.66	0.107	0.0116	0.0036	7.5
14052103	122.6501	29.3834	Spring	E	21.0	8.51	30.76	8.75	1.59	1.61	0.109	0.0127	0.0037	7.6
14052104	122.6501	29.3834	Spring	E	21.0	8.53	30.76	8.87	1.60	1.65	0.111	0.0126	0.0038	7.6
14052105	122.6501	29.3834	Spring	E	21.0	8.50	30.75	8.80	1.56	1.64	0.111	0.0122	0.0037	7.7

DO: dissolved oxygen, COD: chemical oxygen demand, TOC: total organic carbon, TP: total phosphorus, DIN: dissolved inorganic nitrogen, Chl-*a*: chlorophyll *a*.
