# Supplementary material for: Seasonality in Spatial Turnover of Bacterioplankton Along an Ecological Gradient in the East China Sea: Biogeographic Patterns, Processes and Drivers
Source: Microorganisms. 2020 Sep 27;8(10):1484. doi: 10.3390/microorganisms8101484 (PMC7600760; doi:10.3390/microorganisms8101484)
Supplement: Supplementary file 1 [file microorganisms-08-01484-s001.zip › Supplementary Dataset S2.pdf]

Dataset S2 Similarity percentage analysis (SIMPER) showing key driver OTUs responsible for spatial turnover of bacterioplankton in each season.

	OTU ID	Contribution (%)	Cumulative (%)	Taxonomy
Summer (Sep. 2013)	OTU 1	4.368	4.368	D_1__Proteobacteria; D_2__Gammaproteobacteria; D_3__Alteromonadales; D_4__Pseudoalteromonadaceae; D_5__*Pseudoalteromonas*
	OTU 2	3.513	7.881	D_1__Proteobacteria; D_2__Gammaproteobacteria; D_3__Alteromonadales; D_4__Alteromonadaceae; D_5__*Alteromonas* ; D_6__uncultured bacterium
	OTU 3	2.350	10.23	D_1__Proteobacteria; D_2__Alphaproteobacteria; D_3__Rhodobacterales; D_4__Rhodobacteraceae; D_5__uncultured; D_6__uncultured bacterium
	OTU 4	2.335	12.57	D_1__Proteobacteria; D_2__Alphaproteobacteria; D_3__Rhodobacterales; D_4__Rhodobacteraceae; D_5__uncultured
	OTU 5	2.138	14.70	D_1__Proteobacteria; D_2__Gammaproteobacteria; D_3__Oceanospirillales; D_4__Oceanospirillaceae; D_5__*Neptuniibacter*
	OTU 6	1.820	16.52	D_1__Actinobacteria; D_2__Acidimicrobiia; D_3__Acidimicrobiales; D_4__OM1 clade; D_5__*Candidatus* Actinomarina; D_6__uncultured bacterium
	OTU 7	1.789	18.31	D_1__Proteobacteria; D_2__Gammaproteobacteria; D_3__Oceanospirillales; D_4__Oceanospirillaceae; D_5__*Oceanospirillum* ; Ambiguous_taxa
	OTU 8	1.573	19.89	D_1__Proteobacteria; D_2__Alphaproteobacteria; D_3__Rhodobacterales; D_4__Rhodobacteraceae; D_5__*Sulfitobacter*
	OTU 9	1.495	21.38	D_1__Bacteroidetes; D_2__Flavobacteriia; D_3__Flavobacteriales; D_4__Cryomorphaceae; D_5__*Fluviicola* ; D_6__uncultured bacterium
	OTU 10	1.450	22.83	D_1__Proteobacteria; D_2__Gammaproteobacteria; D_3__Alteromonadales; D_4__Pseudoalteromonadaceae; Ambiguous_taxa
	OTU 11	1.450	24.28	D_1__Proteobacteria; D_2__Gammaproteobacteria; D_3__Alteromonadales; D_4__Pseudoalteromonadaceae; D_5__*Pseudoalteromonas*
	OTU 12	1.442	25.72	D_1__Proteobacteria; D_2__Gammaproteobacteria; D_3__Oceanospirillales; D_4__SAR86 clade; Ambiguous_taxa; Ambiguous_taxa
	OTU 13	1.439	27.16	D_1__Proteobacteria; D_2__Gammaproteobacteria; D_3__Oceanospirillales; D_4__Oceanospirillaceae; D_5__*Neptuniibacter*
	OTU 14	1.311	28.47	D_1__Proteobacteria; D_2__Gammaproteobacteria; D_3__Oceanospirillales; D_4__Oceanospirillaceae; D_5__*Neptuniibacter*
	OTU 15	1.268	29.74	D_1__Proteobacteria; D_2__Gammaproteobacteria; D_3__Alteromonadales; D_4__Alteromonadaceae; D_5__*Marinobacter* ; D_6__*Marinobacter hydrocarbonoclasticus*
	OTU 16	1.253	30.99	D_1__Proteobacteria; D_2__Gammaproteobacteria; D_3__Oceanospirillales; D_4__Oceanospirillaceae; D_5__*Oceanospirillum* ; Ambiguous_taxa
	OTU 17	1.106	32.10	D_1__Proteobacteria; D_2__Gammaproteobacteria; D_3__Alteromonadales; D_4__Alteromonadaceae; D_5__*Marinobacter* ; D_6__uncultured *Marinobacter* sp.
	OTU 18	0.952	33.05	D_1__Proteobacteria; D_2__Gammaproteobacteria; D_3__Alteromonadales; D_4__Pseudoalteromonadaceae; D_5__*Pseudoalteromonas*
	OTU 19	0.724	33.78	D_1__Proteobacteria; D_2__Alphaproteobacteria; D_3__Rhodospirillales; D_4__Rhodospirillaceae; D_5__AEGEAN-169 marine group; D_6__uncultured bacterium
	OTU 20	0.704	34.48	D_1__Bacteroidetes; D_2__Flavobacteriia; D_3__Flavobacteriales; D_4__Flavobacteriaceae; D_5__*Salegentibacter* ; Ambiguous_taxa
Autumn (Oct. 2013)	OTU 6	4.458	4.458	D_1__Actinobacteria; D_2__Acidimicrobiia; D_3__Acidimicrobiales; D_4__OM1 clade; D_5__*Candidatus* Actinomarina; D_6__uncultured bacterium
	OTU 21	3.084	7.542	D_1__Proteobacteria; D_2__Betaproteobacteria; D_3__Methylophilales; D_4__Methylophilaceae; D_5__OM43 clade; D_6__uncultured bacterium
	OTU 22	2.447	9.989	D_1__Proteobacteria; D_2__Alphaproteobacteria; D_3__Rhodobacterales; D_4__Rhodobacteraceae
	OTU 23	1.462	11.45	D_1__Proteobacteria; D_2__Betaproteobacteria; D_3__Methylophilales; D_4__Methylophilaceae; D_5__OM43 clade; D_6__uncultured bacterium
	OTU 19	1.411	12.86	D_1__Proteobacteria; D_2__Alphaproteobacteria; D_3__Rhodospirillales; D_4__Rhodospirillaceae; D_5__AEGEAN-169 marine group; D_6__uncultured bacterium
	OTU 24	1.223	14.09	D_1__Proteobacteria; D_2__Gammaproteobacteria; D_3__Oceanospirillales; D_4__SAR86 clade; Ambiguous_taxa; Ambiguous_taxa
	OTU 12	0.950	15.04	D_1__Proteobacteria; D_2__Gammaproteobacteria; D_3__Oceanospirillales; D_4__SAR86 clade; Ambiguous_taxa; Ambiguous_taxa
	OTU 25	0.883	15.92	D_1__Bacteroidetes; D_2__Flavobacteriia; D_3__Flavobacteriales; D_4__Flavobacteriaceae; D_5__NS5 marine group; D_6__uncultured bacterium
	OTU 26	0.835	16.75	D_1__Proteobacteria; D_2__Gammaproteobacteria; D_3__Oceanospirillales; D_4__SS1-B-06-26
	OTU 27	0.787	17.54	D_1__Proteobacteria; D_2__Gammaproteobacteria; D_3__Oceanospirillales; D_4__Alcanivoracaceae; D_5__*Alcanivorax* ; Ambiguous_taxa
	OTU 28	0.777	18.32	D_1__Actinobacteria; D_2__Acidimicrobiia; D_3__Acidimicrobiales; D_4__OM1 clade; D_5__*Candidatus* Actinomarina; D_6__uncultured bacterium
	OTU 29	0.763	19.08	D_1__Actinobacteria; D_2__Acidimicrobiia; D_3__Acidimicrobiales; D_4__OM1 clade; D_5__*Candidatus* Actinomarina; D_6__uncultured bacterium
	OTU 30	0.739	19.82	D_1__Bacteroidetes; D_2__Flavobacteriia; D_3__Flavobacteriales; D_4__Flavobacteriaceae; D_5__NS5 marine group; D_6__uncultured bacterium
	OTU 31	0.668	20.49	D_1__Chloroflexi; D_2__SAR202 clade; Ambiguous_taxa; Ambiguous_taxa; Ambiguous_taxa; Ambiguous_taxa
	OTU 32	0.567	21.05	D_1__Proteobacteria; D_2__Gammaproteobacteria; D_3__Oceanospirillales; D_4__Halomonadaceae; D_5__*Salinicola*
	OTU 33	0.555	21.61	D_1__Proteobacteria; D_2__Betaproteobacteria; D_3__Hydrogenophilales; D_4__Hydrogenophilaceae; D_5__uncultured
	OTU 2	0.543	22.15	D_1__Proteobacteria; D_2__Gammaproteobacteria; D_3__Alteromonadales; D_4__Alteromonadaceae; D_5__*Alteromonas* ; D_6__uncultured bacterium
	OTU 34	0.538	22.69	D_1__Marinimicrobia (SAR406 clade); D_2__uncultured marine bacterium; D_3__uncultured marine bacterium; D_4__uncultured marine bacterium; D_5__uncultured marine bacterium; D_6__uncultured marine bacterium
	OTU 35	0.537	23.23	D_1__Proteobacteria; D_2__Gammaproteobacteria; D_3__E01-9C-26 marine group; Ambiguous_taxa; Ambiguous_taxa; Ambiguous_taxa

	OTU 36	0.531	23.76	D_1__Nitrospinae; D_2__Nitrospinia; D_3__Nitrospinales; D_4__Nitrospinaceae; D_5__uncultured
Winter (Jan. 2014)	OTU 23	5.031	5.031	D_1__Proteobacteria; D_2__Betaproteobacteria; D_3__Methylophilales; D_4__Methylophilaceae; D_5__OM43 clade; D_6__uncultured bacterium
	OTU 37	2.461	7.491	D_1__Proteobacteria; D_2__Gammaproteobacteria; D_3__Cellvibrionales; D_4__Cellvibrionaceae; D_5__*Gilvimarinus* ; Ambiguous_taxa
	OTU 38	2.187	9.678	D_1__Proteobacteria; D_2__Gammaproteobacteria; D_3__Alteromonadales; D_4__Alteromonadaceae; D_5__*Salinimonas* ; Ambiguous_taxa
	OTU 6	1.817	11.50	D_1__Actinobacteria; D_2__Acidimicrobiia; D_3__Acidimicrobiales; D_4__OM1 clade; D_5__*Candidatus* Actinomarina; D_6__uncultured bacterium
	OTU 39	1.496	12.99	D_1__Proteobacteria; D_2__Alphaproteobacteria; D_3__SAR11 clade; D_4__Surface 1; Ambiguous_taxa; Ambiguous_taxa
	OTU 40	1.408	14.40	D_1__Proteobacteria; D_2__Alphaproteobacteria; D_3__SAR11 clade; D_4__Surface 1; D_5__uncultured bacterium; D_6__uncultured bacterium
	OTU 21	1.237	15.64	D_1__Proteobacteria; D_2__Betaproteobacteria; D_3__Methylophilales; D_4__Methylophilaceae; D_5__OM43 clade; D_6__uncultured bacterium
	OTU 41	1.173	16.81	D_1__Proteobacteria; D_2__Alphaproteobacteria; D_3__SAR11 clade; D_4__Surface 1; D_5__uncultured marine bacterium; D_6__uncultured marine bacterium
	OTU 42	0.936	17.75	D_1__Bacteroidetes; D_2__Flavobacteriia; D_3__Flavobacteriales; D_4__Flavobacteriaceae; D_5__*Salegentibacter*
	OTU 20	0.882	18.63	D_1__Bacteroidetes; D_2__Flavobacteriia; D_3__Flavobacteriales; D_4__Flavobacteriaceae; D_5__*Salegentibacter* ; Ambiguous_taxa
	OTU 43	0.790	19.42	D_1__Proteobacteria; D_2__Alphaproteobacteria; D_3__SAR11 clade; D_4__Surface 1; D_5__uncultured bacterium; D_6__uncultured bacterium
	OTU 22	0.769	20.19	D_1__Proteobacteria; D_2__Alphaproteobacteria; D_3__Rhodobacterales; D_4__Rhodobacteraceae
	OTU 44	0.623	20.81	D_1__Proteobacteria; D_2__Alphaproteobacteria; D_3__Rhodobacterales; D_4__Rhodobacteraceae; Ambiguous_taxa; Ambiguous_taxa
	OTU 45	0.578	21.39	D_1__Proteobacteria; D_2__Alphaproteobacteria; D_3__SAR11 clade; D_4__Surface 1
	OTU 19	0.560	21.95	D_1__Proteobacteria; D_2__Alphaproteobacteria; D_3__Rhodospirillales; D_4__Rhodospirillaceae; D_5__AEGEAN-169 marine group; D_6__uncultured bacterium
	OTU 46	0.547	22.49	D_1__Proteobacteria; D_2__AEGEAN-245; Ambiguous_taxa; Ambiguous_taxa; Ambiguous_taxa; Ambiguous_taxa
	OTU 47	0.540	23.03	D_1__Proteobacteria; D_2__Gammaproteobacteria; D_3__Oceanospirillales; D_4__Halomonadaceae; D_5__*Halomonas* ; D_6__uncultured Halomonas sp.
	OTU 48	0.495	23.53	D_1__Proteobacteria; D_2__Alphaproteobacteria; D_3__SAR11 clade; D_4__Surface 1
	OTU 29	0.464	23.99	D_1__Actinobacteria; D_2__Acidimicrobiia; D_3__Acidimicrobiales; D_4__OM1 clade; D_5__*Candidatus* Actinomarina; D_6__uncultured bacterium
	OTU 49	0.457	24.45	D_1__Cyanobacteria; D_2__Cyanobacteria; D_3__SubsectionI; D_4__FamilyI; D_5__*Synechococcus* ; D_6__uncultured bacterium
Spring (May 2014)	OTU 50	8.356	8.356	D_1__Actinobacteria; D_2__Acidimicrobiia; D_3__Acidimicrobiales; D_4__OM1 clade; D_5__*Candidatus* Actinomarina
	OTU 51	3.429	11.79	D_1__Actinobacteria; D_2__Acidimicrobiia; D_3__Acidimicrobiales; D_4__OM1 clade; D_5__*Candidatus* Actinomarina; Ambiguous_taxa
	OTU 40	3.356	15.14	D_1__Proteobacteria; D_2__Alphaproteobacteria; D_3__SAR11 clade; D_4__Surface 1; D_5__uncultured bacterium; D_6__uncultured bacterium
	OTU 6	2.776	17.92	D_1__Actinobacteria; D_2__Acidimicrobiia; D_3__Acidimicrobiales; D_4__OM1 clade; D_5__*Candidatus* Actinomarina; D_6__uncultured bacterium
	OTU 52	2.555	20.47	D_1__Proteobacteria; D_2__Gammaproteobacteria; D_3__Cellvibrionales; D_4__Halieaceae; D_5__OM60(NOR5) clade
	OTU 53	2.134	22.61	D_1__Proteobacteria; D_2__Alphaproteobacteria; D_3__Rhizobiales; D_4__Rhodobiaceae; D_5__*Rhodobium*
	OTU 12	1.795	24.40	D_1__Proteobacteria; D_2__Gammaproteobacteria; D_3__Oceanospirillales; D_4__SAR86 clade; Ambiguous_taxa; Ambiguous_taxa
	OTU 54	1.474	25.88	D_1__Bacteroidetes; D_2__Flavobacteriia; D_3__Flavobacteriales; D_4__Flavobacteriaceae; D_5__*Formosa* ; Ambiguous_taxa
	OTU 23	1.391	27.27	D_1__Proteobacteria; D_2__Betaproteobacteria; D_3__Methylophilales; D_4__Methylophilaceae; D_5__OM43 clade; D_6__uncultured bacterium
	OTU 55	1.365	28.63	D_1__Bacteroidetes; D_2__Flavobacteriia; D_3__Flavobacteriales; D_4__Cryomorphaceae; D_5__uncultured; D_6__uncultured Bacteroidetes bacterium
	OTU 56	1.344	29.98	D_1__Proteobacteria; D_2__Gammaproteobacteria; D_3__Oceanospirillales; D_4__SAR86 clade; D_5__uncultured bacterium; D_6__uncultured bacterium
	OTU 57	1.296	31.27	D_1__Bacteroidetes; D_2__Flavobacteriia; D_3__Flavobacteriales; D_4__Cryomorphaceae; D_5__*Fluviicola* ; D_6__uncultured bacterium
	OTU 58	1.183	32.45	D_1__Proteobacteria; D_2__Gammaproteobacteria; D_3__Cellvibrionales; D_4__Halieaceae; D_5__OM60(NOR5) clade; D_6__uncultured bacterium
	OTU 59	1.173	33.63	D_1__Bacteroidetes; D_2__Flavobacteriia; D_3__Flavobacteriales; D_4__Flavobacteriaceae; D_5__NS5 marine group
	OTU 39	1.035	34.66	D_1__Proteobacteria; D_2__Alphaproteobacteria; D_3__SAR11 clade; D_4__Surface 1; Ambiguous_taxa; Ambiguous_taxa
	OTU 60	0.949	35.61	D_1__Firmicutes; D_2__Bacilli; D_3__Bacillales; D_4__Family XII; D_5__*Exiguobacterium*
	OTU 48	0.908	36.52	D_1__Proteobacteria; D_2__Alphaproteobacteria; D_3__SAR11 clade; D_4__Surface 1
	OTU 61	0.867	37.39	D_1__Proteobacteria; D_2__Alphaproteobacteria; D_3__Rhodobacterales; D_4__Rhodobacteraceae
	OTU 62	0.849	38.24	D_1__Marinimicrobia (SAR406 clade); Ambiguous_taxa; Ambiguous_taxa; Ambiguous_taxa; Ambiguous_taxa; Ambiguous_taxa
	OTU 63	0.838	39.07	D_1__Bacteroidetes; D_2__Flavobacteriia; D_3__Flavobacteriales; D_4__Cryomorphaceae; D_5__*Fluviicola* ; D_6__uncultured bacterium

key driver OTUs: top 20 OTUs most contributing to the compositional variation of bacterial communities.
