# Supplementary material for: Seasonality in Spatial Turnover of Bacterioplankton Along an Ecological Gradient in the East China Sea: Biogeographic Patterns, Processes and Drivers
Source: Microorganisms. 2020 Sep 27;8(10):1484. doi: 10.3390/microorganisms8101484 (PMC7600760; doi:10.3390/microorganisms8101484)
Supplement: Supplementary file 1 [file microorganisms-08-01484-s001.zip › Supplementary Figures and Tables.pdf]

Supplementary Figures and Tables

Seasonality in spatial turnover of bacterioplankton along an ecological gradient in the East China Sea: biogeographic patterns, processes and drivers

Hanjing Hu[1, 2, 3,†], Jiaying He[2, 3,†], Huizhen Yan[2], Dandi Hou[2], Demin Zhang[1, 2, 3], Lian Liu[4,*], Kai Wang[1, 2, 3,*]

[1] State Key Laboratory for Managing Biotic and Chemical Threats to the Quality and Safety of Agro-products, Ningbo University, Ningbo 315211, China; hhj2141@163.com (H.H.); zhangdemin@nbu.edu.cn (D.Z.)

[2] School of Marine Sciences, Ningbo University, Ningbo 315211, China; joyhejiaying@outlook.com (J.H.); hzyan1995@163.com (H.Y.); houdandi@nbu.edu.cn (D.H.)

[3] Collaborative Innovation Center for Zhejiang Marine High-efficiency and Healthy Aquaculture, Ningbo, 315211, China

[4] Marine Environmental Monitoring Center of Ningbo, SOA, Ningbo 315012, China

[†] These authors contributed equally to this work.

[*] correspondence: llian75@163.com (L.L.), wangkai@nbu.edu.cn (K.W.)

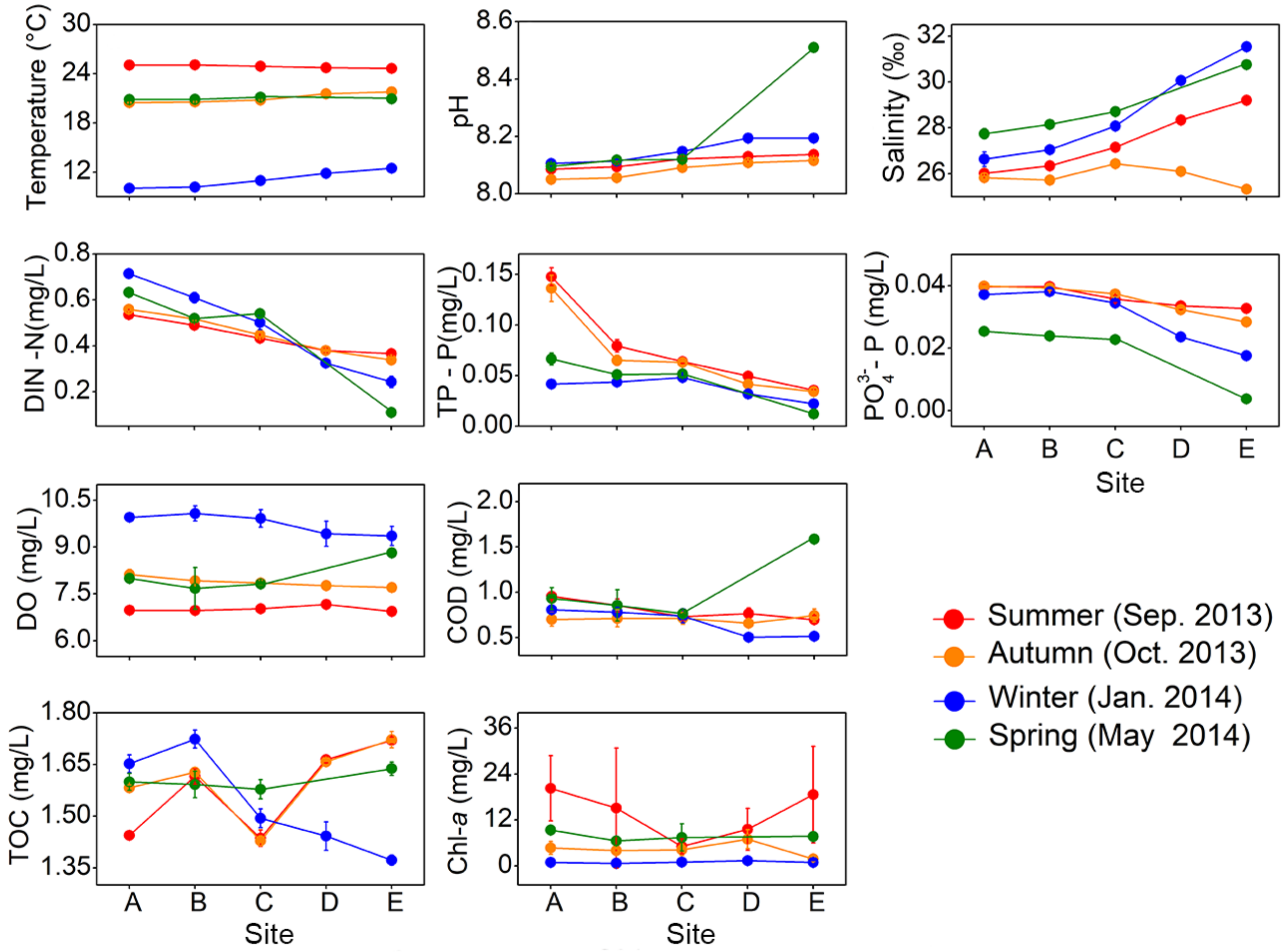

Figure S1. Spatiotemporal dynamics of water environmental parameters across five sites over seasons. Data represent mean ± standard deviation (n = 5). DIN: dissolved inorganic nitrogen; TP: total phosphorus; COD: chemical oxygen demand; DO: dissolved oxygen; TOC: total organic carbon; Chl-*a*: chlorophyll-*a*. Site D was not sampled in spring.

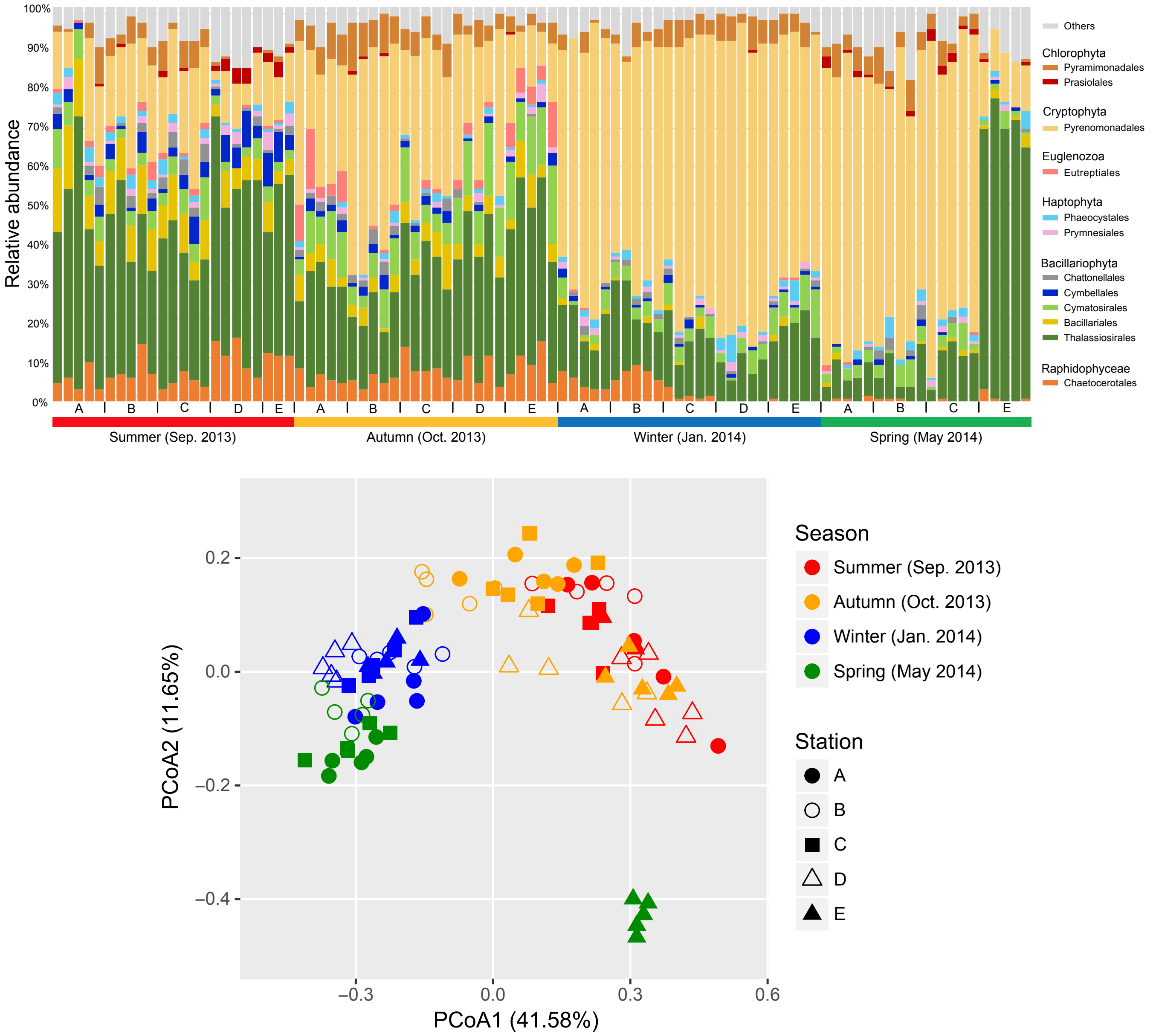

Figure S2. Relative abundance of dominant eukaryotic phytoplankton taxa (upper) and principal coordinate analysis (PCoA) based on Bray-Curtis dissimilarity illustrating the variations in eukaryotic phytoplankton community composition (bottom) based on the Chloroplast 16S rRNA gene data across sites over seasons.

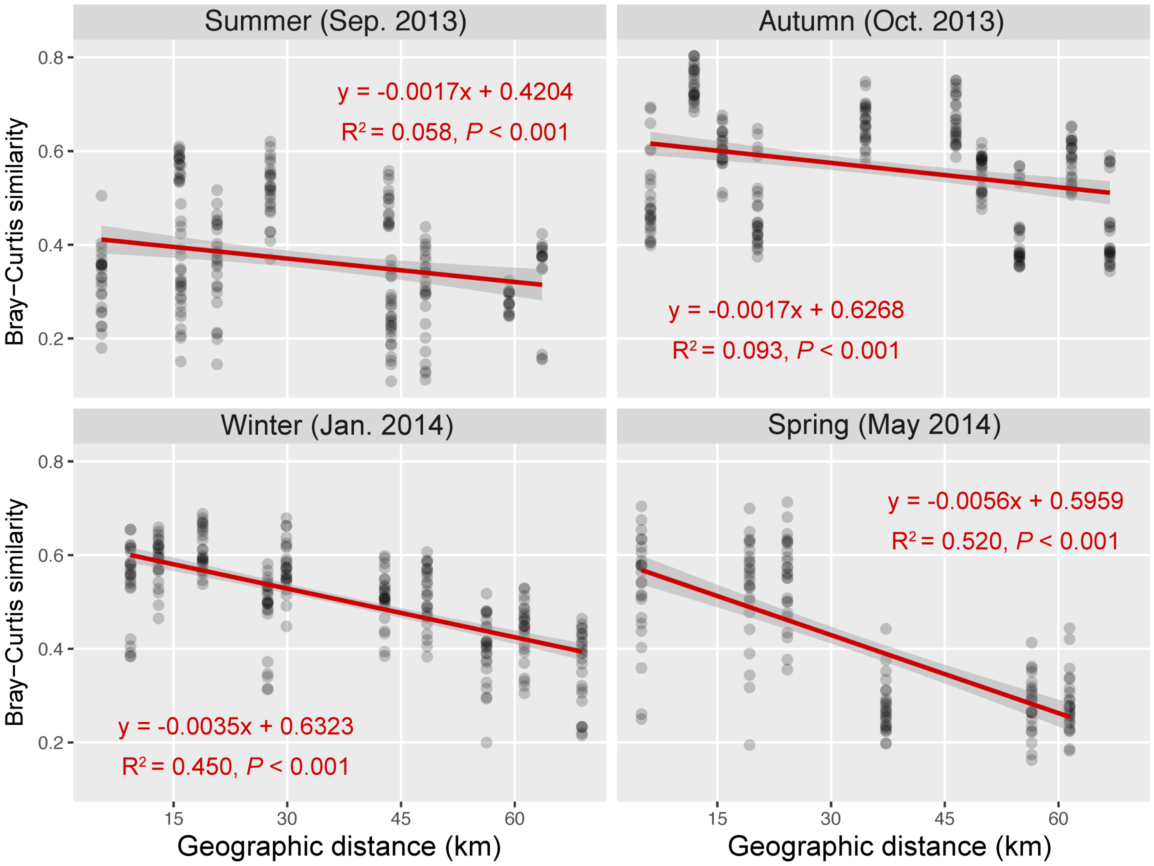

Figure S3. Significant correlations between Bray-Curtis similarity and geographic distance showing the distance-decay pattern in the similarity between bacterial communities. Solid lines present linear fit.

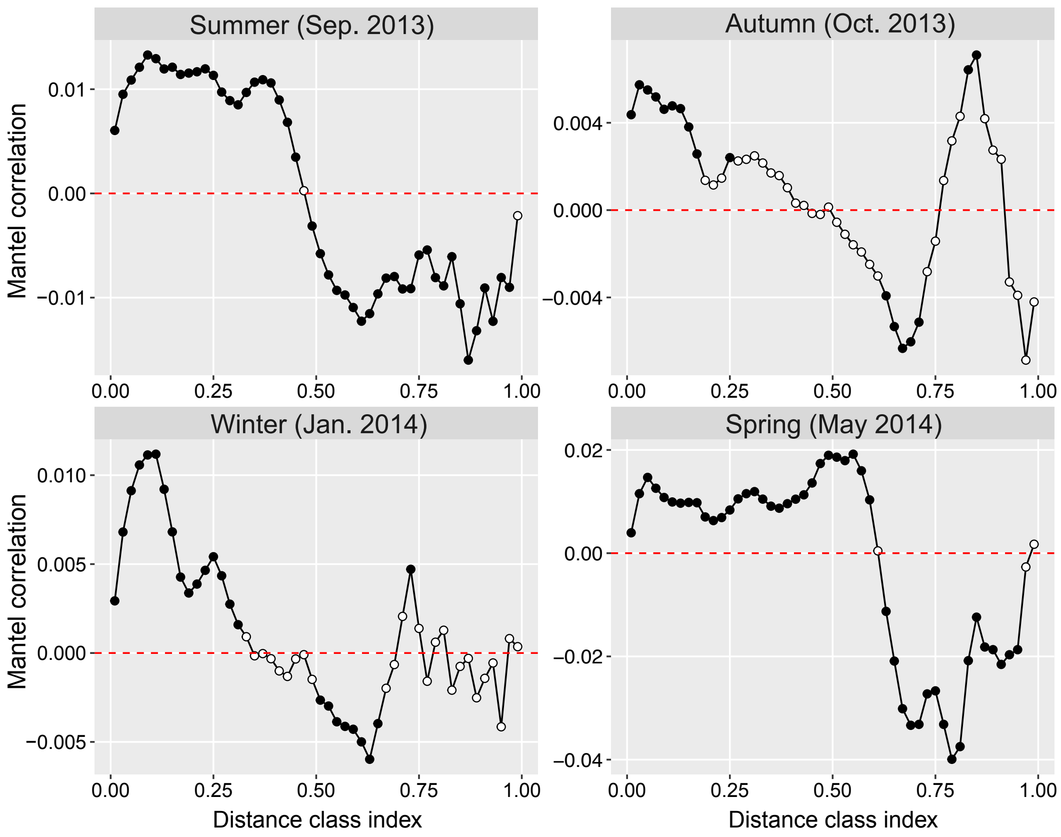

Figure S4. Mantel correlograms testing phylogenetic signal in ecological niches of bacterial OTUs across between-OTU phylogenetic distance classes in four seasons. Solid and open symbols present significant ($P < 0.05$ after Holm correction, 999 permutations) and nonsignificant Pearson correlations of between-OTU niche differences with between-OTU phylogenetic distances, respectively, across phylogenetic distance classes.

Table S1. Two-way crossed analysis of similarity (ANOSIM) of bacterial community composition based on Bray-Curtis dissimilarity across seasons and sampling sites (999 permutations).

Factor	R	*P*
Season		
Global ANOSIM	0.978	0.001
Summer *vs.* Autumn	0.908	0.001
Summer *vs.* Winter	0.944	0.001
Summer *vs.* Spring	0.995	0.001
Autumn *vs.* Winter	1.000	0.001
Summer *vs.* Spring	1.000	0.001
Winter *vs.* Spring	1.000	0.001
Site		
Global ANOSIM	0.627	0.001
Site A *vs.* Site B	0.441	0.001
Site A *vs.* Site C	0.447	0.001
Site A *vs.* Site D	0.785	0.001
Site A *vs.* Site E	0.830	0.001
Site B *vs.* Site C	0.567	0.001
Site B *vs.* Site D	0.964	0.001
Site B *vs.* Site E	0.996	0.001
Site C *vs.* Site D	0.479	0.001
Site C *vs.* Site E	0.781	0.001
Site D *vs.* Site E	0.317	0.002

Table S2. One-way analysis of similarity (ANOSIM) of bacteria community composition based on Bray-Curtis dissimilarity between sites in each season (999 permutations).

	**Summer (Sep. 2013)**			**Autumn (Oct. 2013)**			**Winter (Jan. 2014)**			**Spring (May 2014)**		
	R	P	P_{adj}	R	P	P_{adj}	R	P	P_{adj}	R	P	P_{adj}
A *vs.* B	0.588	0.016	**0.023**	0.424	0.008	**0.009**	0.548	0.008	**0.011**	0.202	0.087	0.131
A *vs.* C	0.548	0.008	**0.020**	0.520	0.008	**0.009**	0.660	0.008	**0.011**	0.050	0.341	0.341
A *vs.* D	0.680	0.008	**0.020**	0.760	0.008	**0.009**	0.916	0.008	**0.011**	—	—	—
A *vs.* E	0.508	0.018	**0.023**	0.740	0.008	**0.009**	0.968	0.008	**0.011**	0.992	0.008	**0.016**
B *vs.* C	0.876	0.008	**0.020**	0.884	0.008	**0.009**	0.380	0.048	0.053	0.128	0.159	0.191
B *vs.* D	1.000	0.008	**0.020**	1.000	0.008	**0.009**	0.892	0.008	**0.011**	—	—	—
B *vs.* E	1.000	0.018	**0.023**	1.000	0.008	**0.009**	1.000	0.008	**0.011**	0.984	0.008	**0.016**
C *vs.* D	0.442	0.024	**0.027**	0.508	0.008	**0.009**	0.488	0.016	**0.020**	—	—	—
C *vs.* E	0.703	0.018	**0.023**	0.532	0.008	**0.009**	0.876	0.008	**0.011**	0.988	0.008	**0.016**
D *vs.* E	0.395	0.071	0.071	0.360	0.008	**0.009**	0.224	0.103	0.103	—	—	—

P_{adj}: P value adjusted by FDR. P_{adj} values in bold indicate significant difference between sites ($P_{adj} < 0.05$).

Table S3. Distance-based multivariate linear model (DistLM) of compositional variation of bacterial community (based on Bray-Curtis dissimilarity) against environmental variables with 999 permutations. Marginal tests: each variable was analyzed individually (ignoring other variables); Sequential tests: forward selection of variables, where the proportion of variation explained by each variable added to the model was conditional on the variables previously included in the model.

	Marginal tests				Conditional tests				
Season	Variable	pseudo-F	*P*	Prop (%)	Variable	pseudo-F	*P*	Prop. (%)	Cumulative Prop. (%)
All	Temp.	22.59	**0.001**	19.89	Temp.	22.59	**0.001**	19.89	19.89
	DO	20.68	**0.001**	8.290	PO_4^{3-}	16.23	**0.001**	12.24	32.13
	PO_4^{3-}	12.59	**0.001**	10.39	DIN	15.47	**0.001**	10.05	42.18
	Salinity	10.54	**0.001**	18.52	pH	12.33	**0.001**	7.110	49.28
	COD	9.167	**0.001**	9.150	Salinity	13.98	**0.001**	7.020	56.31
	pH	8.223	**0.001**	1.950	DTL	5.120	**0.001**	2.460	58.76
	Chl-*a*	7.711	**0.001**	5.480	Chl-*a*	3.685	**0.001**	1.710	60.48
	TP	6.563	**0.001**	6.730	TP	3.471	**0.001**	1.570	62.04
	DTL	5.301	**0.001**	12.15	TOC	1.903	**0.013**	0.850	62.89
	DIN	5.280	**0.001**	7.810	DO	1.332	0.168	0.590	63.49
	TOC	1.805	0.057	5.500	COD	1.105	0.277	0.490	63.98
Summer Sep. 2013	PO_4^{3-}	7.945	**0.001**	27.45	PO_4^{3-}	7.945	**0.001**	27.45	27.45
	Temp.	7.632	**0.001**	26.65	TP	3.486	**0.001**	10.77	38.22
	Salinity	7.106	**0.001**	25.28	DTL	2.408	**0.006**	6.950	45.17
	DIN	7.092	**0.001**	25.24	DIN	2.389	**0.013**	6.420	51.59
	DTL	7.083	**0.001**	25.22	pH	2.135	**0.023**	5.400	56.99
	pH	6.877	**0.001**	24.67	TOC	1.687	0.078	4.100	61.09
	TP	4.580	**0.001**	17.91	Temp.	1.108	0.325	2.660	63.75
	COD	4.561	**0.003**	17.84	DO	1.064	0.374	2.580	66.33
	TOC	2.293	**0.038**	9.840	Chl-*a*	0.852	0.522	2.070	68.40
	Chl-*a*	2.062	0.063	8.940	COD	0.643	0.761	1.610	70.01
	DO	1.866	0.067	8.160	Salinity	0.583	0.791	1.510	71.52
Autumn Oct. 2013	DIN	10.16	**0.001**	30.64	DIN	10.16	**0.001**	30.64	30.64
	pH	9.357	**0.001**	28.92	TP	3.837	**0.001**	10.30	40.94
	TP	9.031	**0.001**	28.19	DO	2.849	**0.002**	7.060	48.00
	DTL	8.899	**0.001**	27.90	Salinity	2.173	**0.004**	5.100	53.10
	Temp.	8.605	**0.001**	27.23	TOC	1.169	0.245	2.720	55.82
	DO	8.339	**0.001**	26.61	COD	1.020	0.400	2.370	58.19
	PO_4^{3-}	7.699	**0.001**	25.08	DTL	0.994	0.469	2.320	60.51
	TOC	1.674	0.134	6.790	PO_4^{3-}	0.950	0.478	2.210	62.72
	Salinity	0.932	0.455	3.900	pH	0.822	0.620	1.940	64.66
	COD	0.655	0.739	2.770	Temp.	0.717	0.740	1.720	66.38
	Chl-*a*	0.584	0.844	2.480	Chl-*a*	0.665	0.825	1.640	68.02
Winter Jan. 2014	DIN	11.12	**0.001**	32.58	DIN	11.12	**0.001**	32.58	32.58
	DTL	10.98	**0.001**	32.31	TP	3.057	**0.001**	8.230	40.81
	pH	10.86	**0.001**	32.06	DTL	2.025	**0.023**	4.970	45.78
	Temp.	10.77	**0.001**	31.90	Salinity	1.710	**0.043**	4.040	49.82
	Salinity	10.64	**0.001**	31.64	pH	1.367	0.136	3.620	53.44
	PO_4^{3-}	9.703	**0.001**	29.67	COD	1.292	0.181	3.370	56.81
	TOC	8.420	**0.001**	26.80	PO_4^{3-}	1.172	0.254	3.030	59.84
	COD	8.138	**0.001**	26.13	DO	1.095	0.342	2.570	62.41
	TP	6.787	**0.001**	22.79	TOC	1.026	0.404	2.410	64.82
	DO	4.831	**0.002**	17.36	Chl-*a*	0.859	0.618	2.030	66.85
	Chl-*a*	1.853	0.061	7.450	Temp.	0.767	0.714	1.850	68.70
Spring May 2014	pH	13.95	**0.001**	43.67	pH	13.95	**0.001**	43.67	43.67
	PO_4^{3-}	13.41	**0.001**	42.69	Chl-*a*	2.954	**0.006**	8.340	52.01

	DIN	12.96	**0.001**	41.85	DTL	1.914	0.053	4.920	56.93
	Salinity	11.75	**0.001**	39.49	PO_4^{3-}	1.677	0.087	4.550	61.48
	COD	11.67	**0.001**	39.34	DIN	1.666	0.096	3.940	65.42
	TP	11.48	**0.001**	38.94	COD	1.541	0.142	3.820	69.24
	DTL	11.29	**0.001**	38.55	Temp.	1.322	0.227	3.030	72.27
	DO	7.423	**0.001**	29.20	TP	1.058	0.355	2.490	74.76
	TOC	3.841	**0.005**	17.58	DO	0.967	0.500	2.230	76.99
	Chl-*a*	1.564	0.165	8.000	Salinity	0.689	0.703	1.640	78.63
	Temp.	0.689	0.618	3.690	TOC	0.452	0.889	1.140	79.77

DTL, distance to land; DIN, dissolved inorganic nitrogen; TP, total phosphorus; Temp., water temperature; COD, chemical oxygen demand; DO, dissolved oxygen; TOC, total organic carbon. Chl-*a*, chlorophyll-*a*. *P* values in bold indicate significant correlations ($P < 0.05$).

Table S4. Spearman's correlation coefficients between geo-environmental factors.

Season	Variables	DTL	Temp.	pH	Salinity	DO	COD	TOC	DIN	TP	PO_4^{3-}
Summer (Sep. 2013)	Temp.	**-0.904*****									
	pH	**0.877*****	**-0.851*****								
	Salinity	**0.979*****	**-0.885*****	**0.867*****							
	DO	0.302	**-0.417***	**0.292***	0.341						
	COD	**-0.777*****	**0.693*****	**-0.822*****	**-0.765*****	-0.134					
	TOC	**0.693*****	**-0.599****	**0.469***	**0.685*****	0.255	-0.332				
	DIN	**-0.971*****	**0.913*****	**-0.828*****	**-0.958*****	-0.380	**0.759*****	**-0.699*****			
	TP	**-0.979*****	**0.874*****	**-0.873*****	**-0.968*****	-0.283	**0.805*****	**-0.668*****	**0.958*****		
	PO_4^{3-}	**-0.882*****	**0.867*****	**-0.842*****	**-0.878*****	-0.270	**0.762*****	**-0.510***	**0.872*****	**0.902*****	
	Chl-*a*	-0.130	0.187	-0.245	-0.152	-0.254	0.259	0.136	0.184	0.121	0.141
Autumn (Oct. 2013)	Temp.	**0.976*****									
	pH	**0.937*****	**0.923*****								
	Salinity	-0.196	-0.194	-0.110							
	DO	**-0.841*****	**-0.805*****	**-0.785*****	0.119						
	COD	0.081	0.016	-0.039	-0.208	-0.031					
	TOC	**0.687*****	**0.670*****	**0.583****	**-0.671*****	**-0.515****	0.027				
	DIN	**-0.981*****	**-0.964*****	**-0.922*****	0.199	**0.889*****	-0.004	**-0.663*****			
	TP	**-0.942*****	**-0.913*****	**-0.861*****	0.320	**0.763*****	0.067	**-0.746*****	**0.931*****		
	PO_4^{3-}	**-0.934*****	**-0.902*****	**-0.888*****	0.165	**0.732*****	-0.040	**-0.671*****	**0.888*****	**0.863*****	
	Chl-*a*	-0.345	-0.339	-0.203	0.508	0.191	**-0.474***	-0.365	0.323	0.293	0.294
Winter (Jan. 2014)	Temp.	**0.984*****									
	pH	**0.931*****	**0.919*****								
	Salinity	**0.969*****	**0.948*****	**0.928*****							
	DO	**-0.639****	**-0.613****	**-0.607****	**-0.630****						
	COD	**-0.837*****	**-0.811*****	**-0.820*****	**-0.831*****	**0.647*****					
	TOC	**-0.868*****	**-0.864*****	**-0.843*****	**-0.900*****	**0.657*****	**0.731*****				
	DIN	**-0.981*****	**-0.967*****	**-0.931*****	**-0.969*****	**0.671*****	**0.840*****	**0.868*****			
	TP	**-0.600****	**-0.597****	**-0.557****	**-0.589****	**0.645*****	**0.648*****	**0.620*****	**0.602****		
	PO_4^{3-}	**-0.907*****	**-0.897*****	**-0.877*****	**-0.894*****	**0.657*****	**0.845*****	**0.943*****	**0.907*****	**0.654*****	
	Chl-*a*	0.258	0.245	0.341	0.237	-0.335	-0.337	-0.363	-0.254	-0.187	-0.369
Spring (May 2014)	Temp.	**0.733*****									
	pH	**0.898*****	**0.558***								
	Salinity	**0.964*****	**0.710*****	**0.841*****							
	DO	0.272	-0.231	0.312	0.227						
	COD	0.365	-0.151	0.336	0.417	**0.628****					
	TOC	0.359	-0.117	0.398	0.410	0.431	**0.573****				
	DIN	**-0.776*****	-0.271	**-0.792*****	**-0.748*****	-0.399	**-0.484***	**-0.468***			
	TP	**-0.877*****	**-0.503***	**-0.900*****	**-0.847*****	-0.263	-0.423	**-0.500***	**0.879*****		
	PO_4^{3-}	**-0.941*****	**-0.734*****	**-0.867*****	**-0.917*****	-0.242	-0.336	-0.362	**0.711*****	**0.836*****	
	Chl-*a*	-0.319	-0.287	**-0.491***	-0.274	0.293	0.270	0.134	0.408	**0.521***	0.375

DTL, distance to land; DIN, dissolved inorganic nitrogen; TP, total phosphorus; Temp., water temperature; COD, chemical oxygen demand; DO, dissolved oxygen; TOC, total organic carbon. Chl-*a*, chlorophyll-*a*. Bold values indicate significant correlations ($^{*}P < 0.05$; $^{**}P < 0.01$; $^{***}P < 0.001$).
